# Electronic Structure of Metallophlorins: Lessons from Iridium and Gold Phlorin Derivatives

**DOI:** 10.1021/acs.inorgchem.4c00483

**Published:** 2024-05-14

**Authors:** Simon Larsen, Joseph A. Adewuyi, Kolle E. Thomas, Jeanet Conradie, Yoann Rousselin, Gaël Ung, Abhik Ghosh

**Affiliations:** † Department of Chemistry, University of Tromsø, N-9037 Tromsø, Norway; ‡ Department of Chemistry, University of Connecticut, 55 N. Eagleville Rd, Storrs, Connecticut 06269, United States; § Department of Chemistry, University of the Free State, P.O. Box 339, Bloemfontein 9300, Republic of South Africa; ∥ ICMUB, UMR CNRS 6302, Université Bourgogne Franche-Comte, BP 47870, Dijon Cedex 21078, France

## Abstract

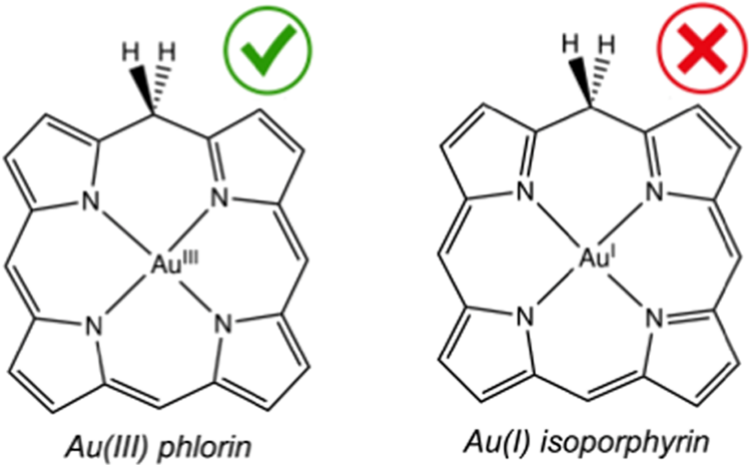

Phlorins have long remained underexplored relative to their fully conjugated counterparts, such as porphyrins, hydroporphyrins, and corroles. Herein, we have attempted to bridge that knowledge gap with a scalar-relativistic density functional theory (DFT) study of unsubstituted iridium and gold phlorin derivatives and a multitechnique experimental study of iridium-bispyridine and gold complexes of 5,5-dimethyl-10,15,20-tris(pentafluorophenyl)phlorin. Theory and experiments concur that the phlorin derivatives exhibit substantially smaller HOMO–LUMO gaps, as reflected in a variety of observable properties. Thus, the experimentally studied Ir and Au complexes absorb strongly in the near-infrared (NIR), with absorption maxima at 806 and 770 nm, respectively. The two complexes are also weakly phosphorescent with emission maxima at 950 and 967 nm, respectively. They were also found to photosensitize singlet oxygen formation, with quantum yields of 40 and 28%, respectively. The near-infrared (NIR) absorption and emission are consonants with smaller electrochemical HOMO–LUMO gaps of ∼1.6 V, compared to values of ∼2.1 V, for electronically innocent porphyrins and corroles. Interestingly, both the first oxidation and reduction potentials of the Ir complex are some 600 mV shifted to more negative potentials relative to those of the Au complex, indicating an exceptionally electron-rich macrocycle in the case of the Ir complex.

## Abstract 2

A multitechnique study on gold and iridium phlorins has significantly deepened our understanding of these less-studied macrocycles. Metallophlorins are strongly ruffled, albeit with a low inversion barrier. With low HOMO−LUMO gaps, the complexes exhibit strong NIR absorption and weak NIR phosphorescence, as well as modest singlet oxygen sensitization efficiencies. For the gold complexes, DFT calculations indicate an Au(III) phlorin, as opposed to an Au(I) isoporphyrin, description.

## Introduction

Since the beginnings of photodynamic therapy in the early part of the twentieth century, porphyrins have been a cornerstone of the approach.^1,2^ While many of the classic photosensitizers are based on free-base porphyrins and hydroporphyrins,^3,4^ triplet photosensitizers, such as 5d metalloporphyrins and metallocorroles, have attracted substantial attention in recent years.^5−7^ Very recently, we have begun to survey a wider range of 4d and 5d metalloporphyrin analogues, with an emphasis on near-infrared (NIR) absorbing systems. Examples include recent studies of isocorroles^8,9^ and 6-azahemiporphycenes.^10^ In the present study, we have attempted to extend our survey to phlorins,^11−14^ a class of reduced porphyrins with a storied history.

In 1960, Woodward and co-workers discovered a *dihydroporphyrin* intermediate, which readily oxidized to a porphyrin, in the course of their celebrated total synthesis of chlorophyll a.^15−17^ Woodward named this intermediate phlorin and speculated that a similar isomer of porphyrin should also exist. Ten years later, Dolphin and co-workers realized Woodward’s prediction by isolating a zinc isoporphyrin.^18^ Over the subsequent half-century, several examples of phlorins^11−14^ and isoporphyrins^19−22^ have appeared in the literature. In particular, phlorins have been recognized as intermediates in the proton-coupled electron transfer (PCET)-based oxidation of porphyrinogens to porphyrins.^23−26^ However, our general appreciation of the electronic structure of phlorins has languished, a key knowledge gap that we have also sought to bridge in this study.

**Scheme 1:**
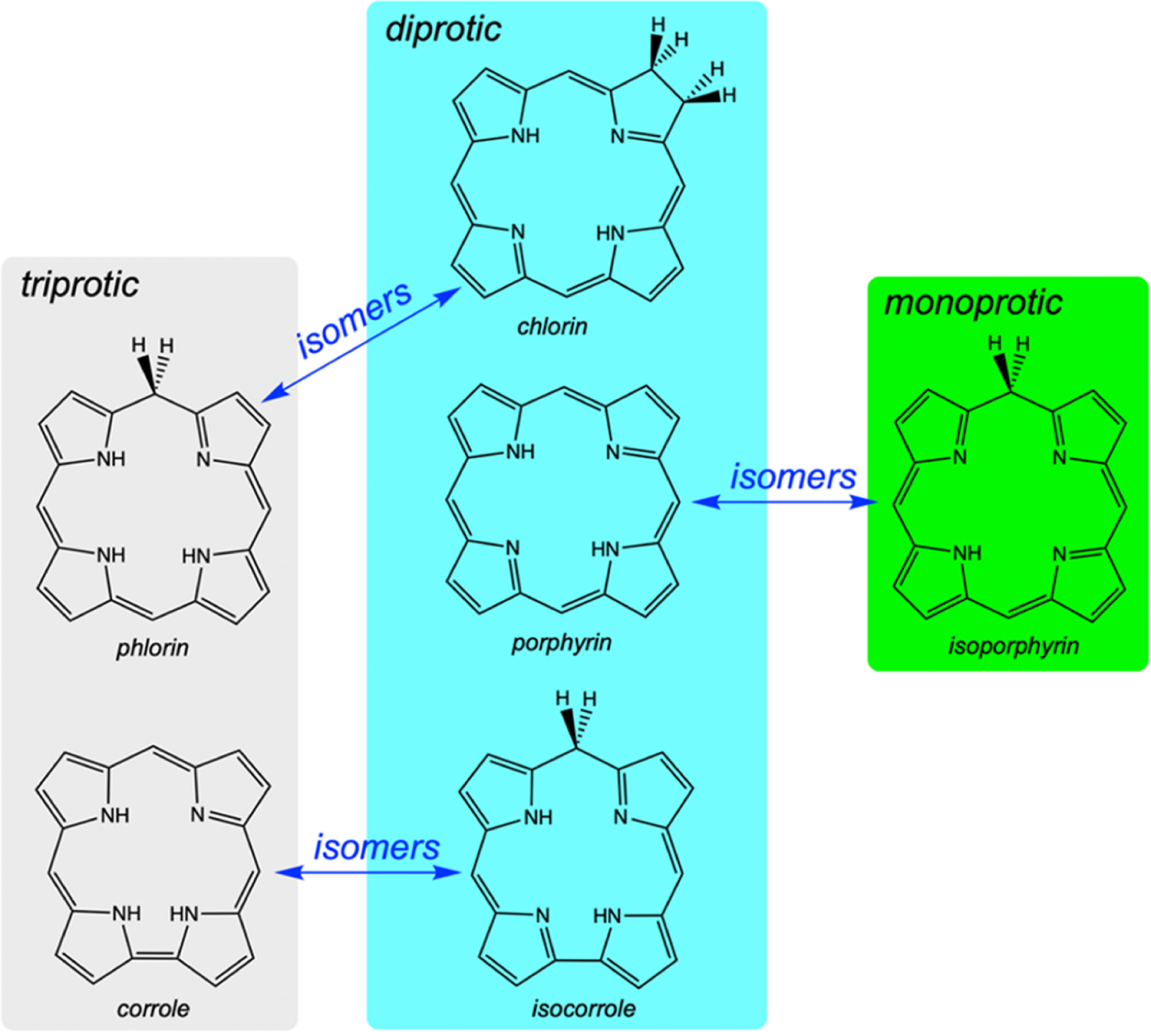
Proticity and Isomeric Relationships Among Free-Base Porphyrinoids

In free-base form, the distinction between phlorin and isoporphyrin is rather obvious (Scheme 1). Phlorin is a two-electron reduced derivative of porphyrin, in fact an isomer of chlorin. Phlorin also resembles corrole by virtue of its triprotic N_4_ core. Isoporphyrin, in contrast, is a porphyrin isomer with a monoprotic N_4_ core. For complexes involving redox-active metals, however, the distinction between the two macrocycles can be tricky; indeed, an Au(III) phlorin and an Au(I) isoporphyrin are only valence tautomers. Metal–ligand bond distances provide an important clue, but quantum chemistry, with careful control of group theory, can also provide a definitive distinction, as we show in this study.

**Scheme 2:**
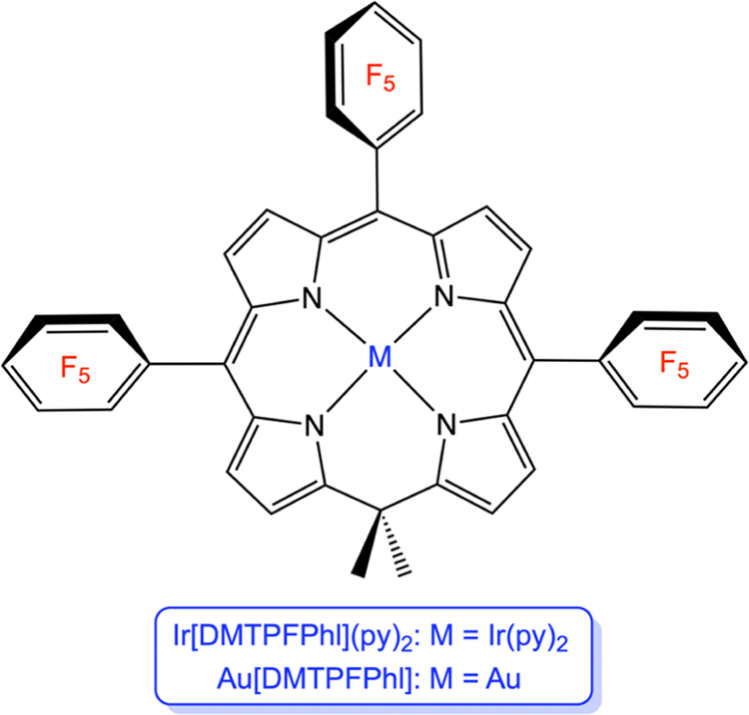
Phlorin Derivatives Experimentally Studied in This Work

Against that backdrop, we report here a combined experimental and theoretical study of two 5d metallophlorins, including wide-ranging density functional theory (DFT) calculations, temperature-dependent NMR studies of structure and conformation, cyclic voltammetry, optical and photophysical studies, and singlet oxygen sensitization measurements. The experimental studies were carried out on gold 5,5-dimethyl-10,15,20-tris(pentafluorophenyl)phlorin, Au[DMTPFPhl], a known compound,^27,28^ and on its iridium-bispyridine analogue Ir[DMTPFPhl](py)_2_, a new compound and the first iridium phlorin (Scheme 2). The results, in our view, significantly deepen our understanding of key geometric and electronic structural aspects of metallophlorins.

## Results and Discussion

### DFT Calculations

**Figure 1:**
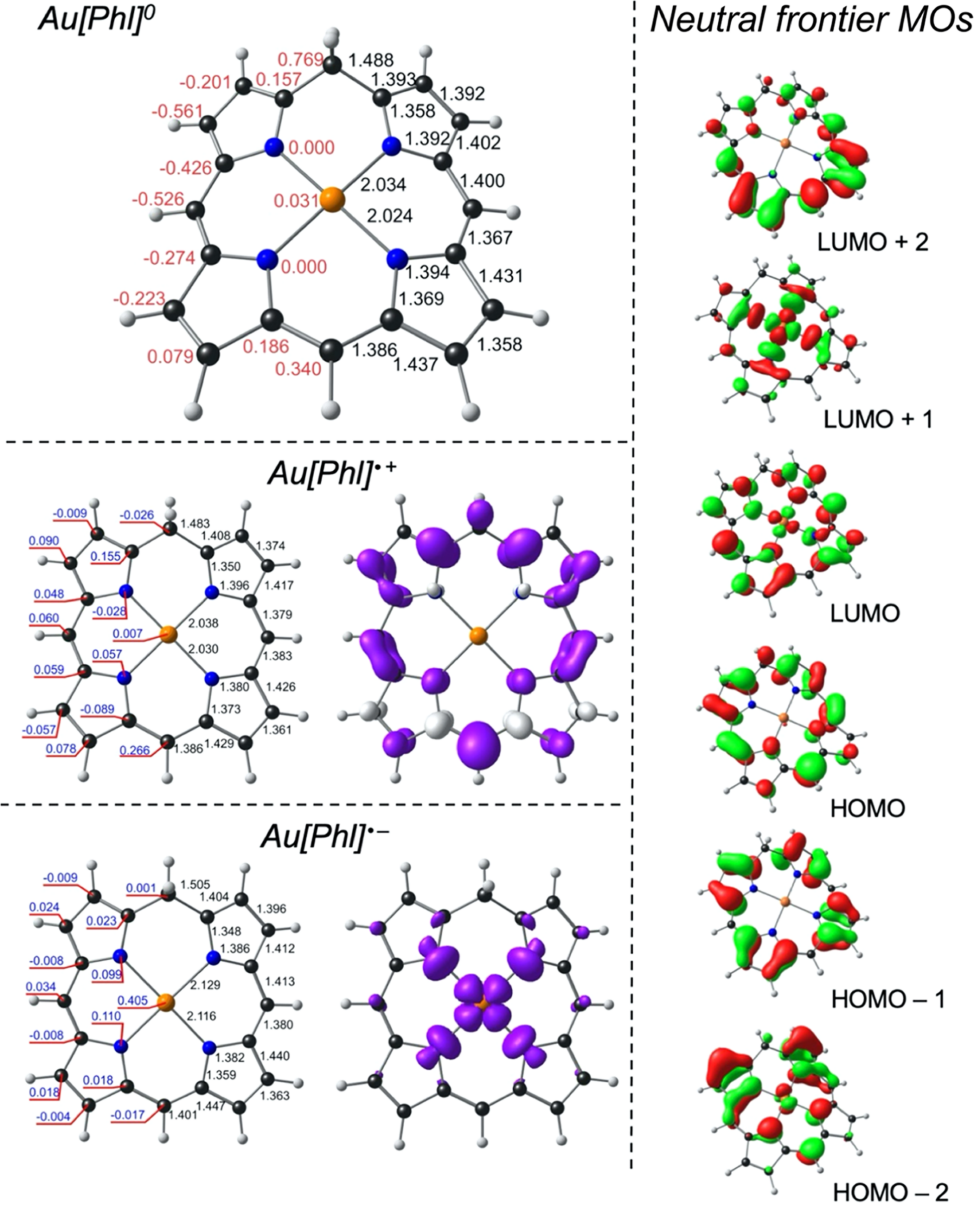
B3LYP-D3/STO-TZ2P results for Au[Phl]. Top left: Optimized bond distances (Å, in black) and displacements from the N_4_ plane (Å, in red) for neutral Au[Phl]. Bottom middle and left: Optimized bond distances (Å, in black) and Mulliken spin populations (in blue) for Au[Phl] cations and anions. Right: Selected Kohn–Sham MOs for neutral Au[Phl].

**Figure 2:**
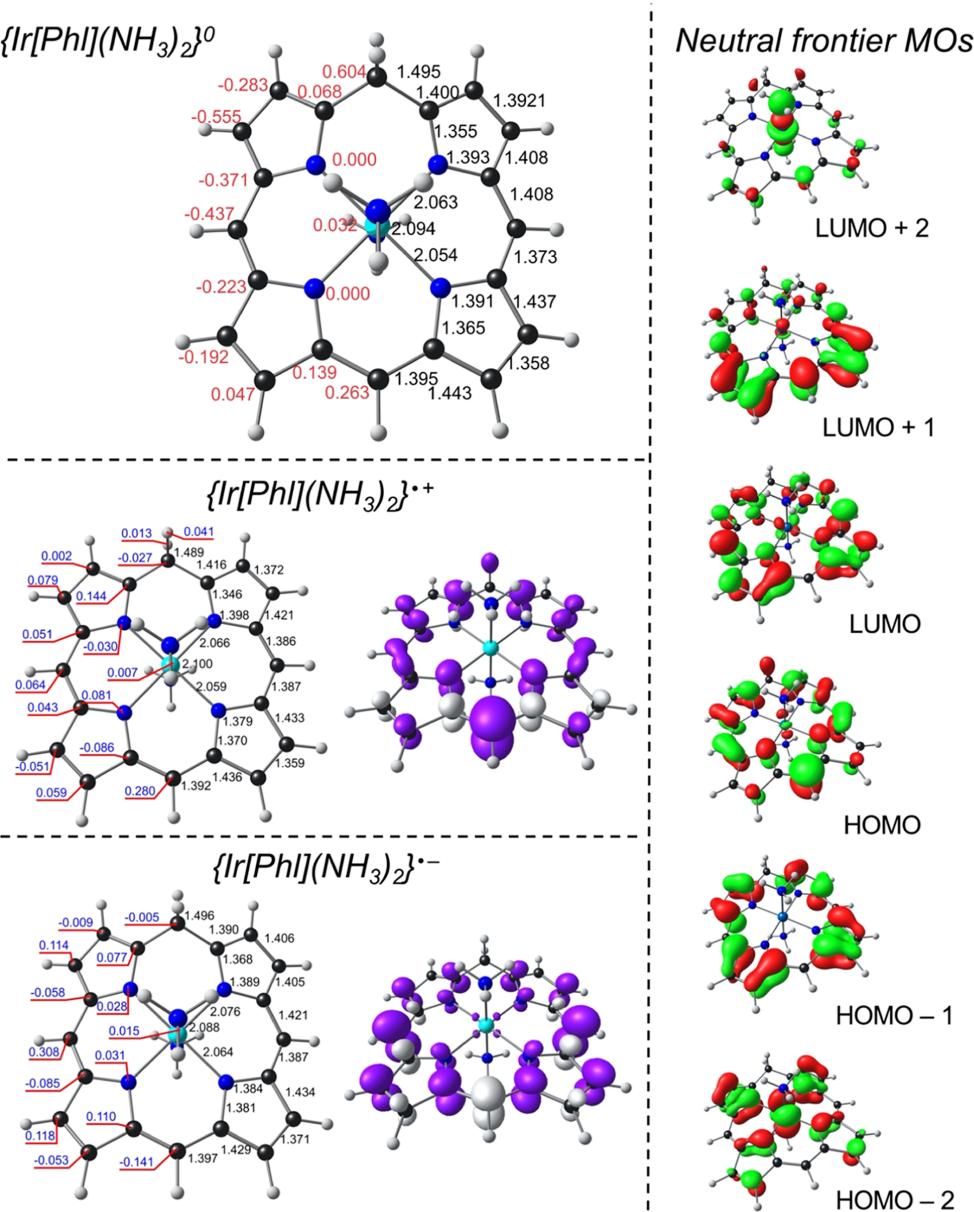
B3LYP-D3/STO-TZ2P results for Ir[Phl](NH_3_)_2_. Top left: Optimized bond distances (Å, in black) and displacements from the N_4_ plane (Å, in red) for neutral Ir[Phl](NH_3_)_2_. Bottom middle and left: Optimized bond distances (Å, in black) and Mulliken spin populations (in blue) for Au[Phl] cations and anions. Right: Selected Kohn–Sham MOs for neutral Ir[Phl](NH_3_)_2_.

Phlorin may be viewed as formally arising via a nucleophilic attack by a hydride on the *meso* carbon of a porphyrin. This formal transformation has profound structural and electronic consequences. Structurally, phlorins are strongly ruffled, with *meso* positions alternately above and below the mean plane of the central nitrogens. The ruffling destroys the σ/π distinction among molecular orbitals, resulting in extensive metal(d)-macrocycle(π) orbital mixing. Other electronic aspects of phlorins may be anticipated from Gouterman’s four-orbital model.^29−31^ Thus, a porphyrin’s a_2u_ HOMO, with large amplitudes at *meso* positions, may be expected to be strongly destabilized in a phlorin, whereas the a_1u_ HOMO should remain relatively unperturbed. These expectations are confirmed by scalar-relativistic DFT (B3LYP^32−34^-D3^35^/STO-TZ2P) calculations on the model compounds Au[Phl] and Ir[Phl](NH_3_)_2_, where Phl^3–^ is the trianion of unsubstituted phlorin. Both molecules were found to exhibit strongly ruffled, *C*_s_ optimized geometries, with the planar *C*_2v_ geometries about a quarter of an eV higher in energy. To place their electronic properties in context, we also performed analogous calculations on unsubstituted platinum porphyrin, Pt[Por], and gold corrole, Au[Cor].^36^ The main results are presented in Table 1 and Figures 1 and 2 and may be summarized as follows.

- For the two reference compounds Pt[Por] and Au[Cor], the two lowest vertical IPs are similar, reflecting near-degenerate HOMOs and consistent with Gouterman’s four-orbital model.^29−31,37^ The values for Pt[Por] are in excellent agreement with those determined for free-base porphine with both gas-phase photoelectron spectroscopy^38^ and DFT calculations.^39−43^ The EAs of about 1.2 eV are also in excellent agreement with experimental gas-phase values^44^ and previous DFT calculations.^45,46^
- The first ionization potential, whether vertical or adiabatic, follows the order Pt[Por] > Au[Cor] > Au[Phl] > Ir[Phl](NH_3_)_2_. In other words, the phlorin derivatives are expected to exhibit substantially lower first IPs than similarly substituted porphyrins or corroles. Their second IPs, however, are rather similar to that of Au[Cor], as expected for ionization of the a_1u_-type HOMO.
- Table 1 indicates a surprisingly large difference in the first ionization potentials of Au[Phl] and Ir[Phl](NH_3_)_2_, with the former exceeding the latter by nearly two-thirds of an eV. Thus, naively speaking, the phlorin in the Ir complex appears to be significantly more electron-rich than that in the Au complex.
- The two metallophlorins also exhibit significantly different electron affinities, with Au[Phl] > Ir[Phl](NH_3_)_2_, once again suggesting a more electron-rich macrocycle in the latter complex. However, the strong ruffling of the macrocycle results in a significant overlap between the phlorin’s π-LUMO and the Au 5d_*x*^2^–*y*^2^_ orbital, so electron addition in the gold case occurs in a significantly (but far from exclusively) metal-centered manner.
- The two phlorin derivatives exhibit much smaller singlet–triplet gaps, relative to Pt[Por] or Au[Cor], reflecting smaller HOMO–LUMO gaps, as expected for nonaromatic compounds.

**Table 1:** All-Electron B3LYP-D3/STO-TZ2P Gas-Phase Properties (eV) of Selected Metallotetrapyrroles, Where Phl, Cor, and Por Denote Unsubstituted Phlorin and Corrole Trianions and the Porphyrin Dianion, Respectively: First and Second Vertical Ionization Potentials (*I*_v1_ and *I*_v2_), Adiabatic Ionization Potentials (*I*_a_), Vertical Electron Affinities (*A*_v_), Adiabatic Electron Affinities (*A*_a_), and Adiabatic Singlet–Triplet Gaps (Δ*E*_S-T_)^a^

property	Au[Phl] (*C*_s_)	Ir[Phl](NH_3_)_2_ (*C*_s_)	Au[Cor] (*C*_2v_)	Pt[Por] (*D*_4h_)
*I*_v1_	5.89 (^2^A′)	5.27 (^2^A′)	6.56 (^2^B_2_)	6.91 (^2^A_1u_)
*I*_v2_	6.96 (^2^A″)	6.48 (^2^A″)	6.68 (^2^A_2_)	7.00 (^2^A_2u_)
*I*_a_	5.78 (^2^A′)	5.15 (^2^A′)	6.50 (^2^B_2_)	6.86^b^
*A*_v_	1.29 (^2^A″)	0.74 (^2^A″)	1.07 (^2^A_2_)	0.90^c^
*A*_a_	1.50 (^2^A″)	0.81 (^2^A″)	1.18 (^2^A_2_)	1.20^b^
Δ*E*_S-T_	0.66 (^3^A″)	0.86 (^3^A″)	1.62 (^3^B_1_)	2.04^b^

a For each value, the symmetry of the higher-energy state is indicated within parentheses (the lower-energy state always being the fully symmetric ground state).

b These states were only optimized under *C*_s_, with the symmetry plane identified with the mean molecular plane, so as to allow for Jahn–Teller distortions.

c Because the LUMOs of Pt[Por] are degenerate (*e*_g_) under *D*_4h_, the vertical electron affinity was computed by running the anion calculation under the Abelian subgroup *D*_2h_ (which has no degenerate representations).

Finally, an examination of the occupied MOs allowed us to rule out any potential isoporphyrin character for the compounds studied. Taking the case of Au[Phl], note that the purely macrocycle-based HOMO transforms as a′ under *C*_s_ point group symmetry, whereas the LUMO and LUMO + 1, each with partial Au(5d_*x*^2^–*y*^2^_) character, transform as a″. Accordingly, using simple group-theoretical manipulations (moving two electrons from a′ to a′′), we could computationally study the Au(I) isoporphyrin state. The optimized geometry revealed a relatively planar macrocycle, as indeed observed experimentally for an authentic Zn-isoporphyrin complex.^47^ However, B3LYP-D3 calculations (as well as DFT calculations with several other common exchange–correlation functionals) revealed an energy ∼40 kcal/mol above the Au(III) phlorin ground state, conclusively ruling out an isoporphyrin ground state. Whether axial ligands, such as phosphines and isocyanides, might stabilize an Au(I) isoporphyrin remains an intriguing question for the future.

The above theoretical findings are nicely confirmed by Ultraviolet–visible–near-infrared (UV–vis-NIR) absorption, phosphorescence, and electrochemical measurements, as outlined below.

### Synthesis and Characterization

**Figure 3:**
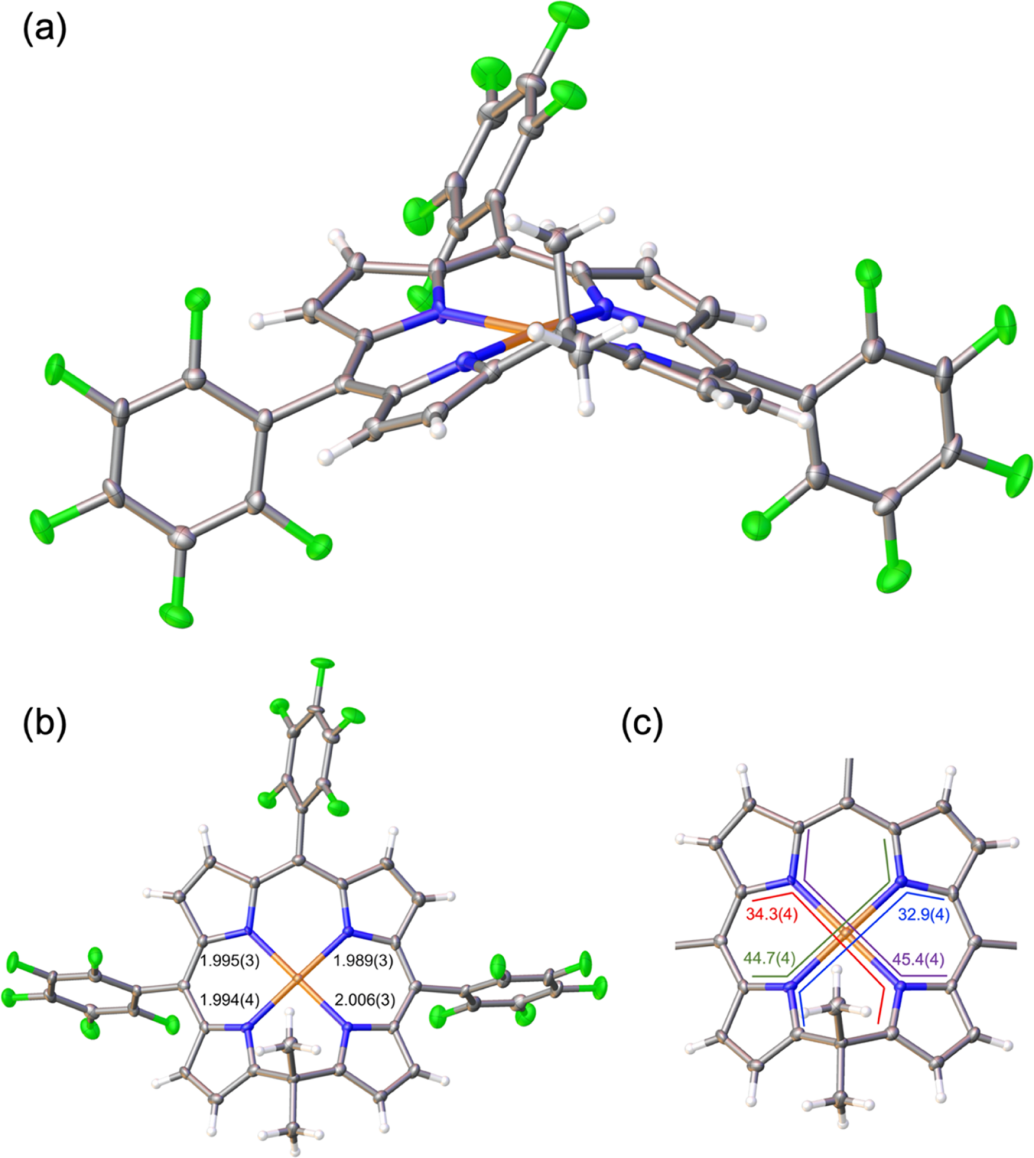
X-ray structure of Au[DMTPFPhl] with 50% thermal ellipsoids (a). Selected bond distances (Å) and dihedrals (°) are indicated in panels (b) and (c).

We chose an iridium-bispyridine and a gold phlorin derivative for a general physicochemical study of metallophlorins. These complexes are not only expected to be electronically innocent but were also of interest as potential candidates for NIR phosphorescence and singlet oxygen-sensitizing properties. The ligand used in our experimental studies, 5,5-dimethyl-10,15,20-tris(pentafluorophenyl)phlorin, DMTPFPhl, was chosen in light of the superior photophysical properties found for pentafluorophenyl-appended corroles.^48^ Of the two complexes, Au[DMTPFPhl] is a known compound;^28^ its crystal structure (Figure 3), however, had not been previously reported. The complex, like other phlorins, exhibits a strongly ruffled macrocycle. The ^1^H and ^19^F NMR spectra of the Ir complex from ambient temperature down to −50 °C (Figures S1 and S2), however, are consistent with time-averaged *C*_2v_, as opposed to *C*_s_, symmetry. Thus, the two geminal methyl groups at the 5-position appear as a single signal. Also, in the ^19^F NMR spectra, the *ortho* and *meta* fluorines on a given *meso*-pentafluorophenyl group do not split into diastereotopic pairs. B3LYP-D3 calculations suggest a plausible explanation for the conundrum: rapid ruffling inversion of the phlorin macrocycle on the nuclear magnetic resonance (NMR) time scale. Thus, the calculations predict an energy of only 0.18 eV for the planar *C*_2v_ conformation of Ir[Phl](NH_3_)_2_ relative to the ruffled *C*_s_ ground state (the corresponding value for Au[Phl] is 0.24 eV). Frequency analyses established these planar forms as true transition states with a single imaginary frequency. We also calculated the barriers for the actual complexes studied, including all peripheral substituents, and found the barriers to be only slightly higher, ∼0.33 eV for either metal; such a value is also consistent with time-averaged *C*_2v_ geometry on the NMR time scale.

**Figure 4:**
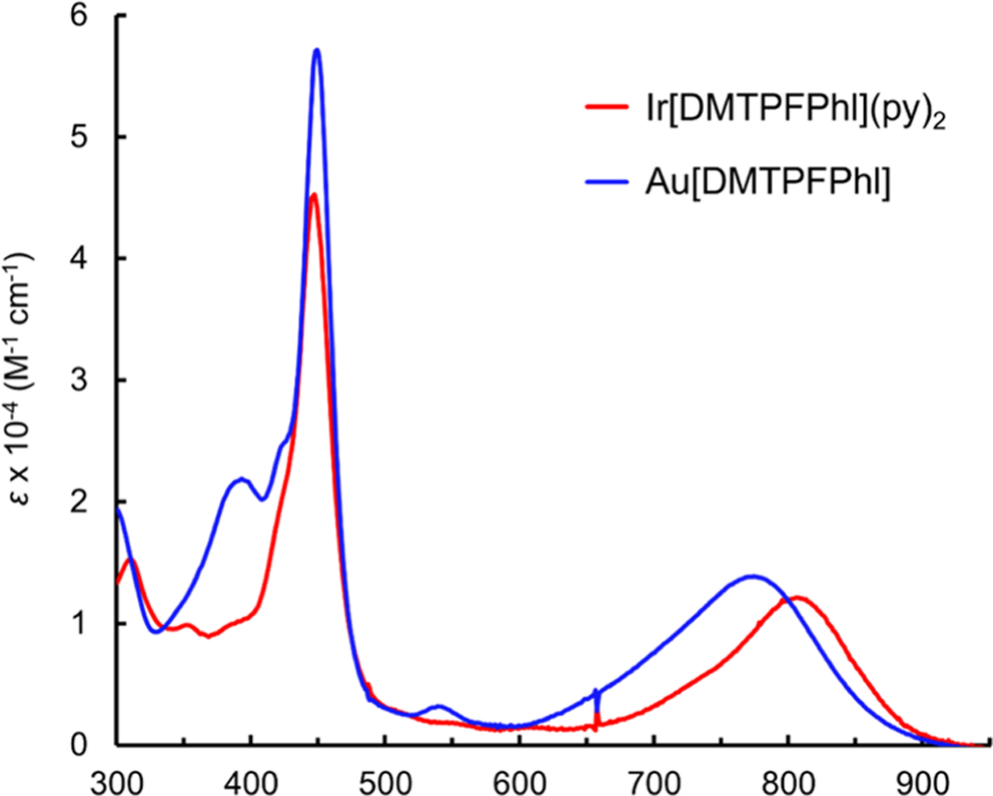
UV–vis-NIR spectra in dichloromethane at ambient temperature.

As noted before for Au phlorins,^28^ both complexes exhibit strong NIR absorption in the 700–900 nm region, with λ_max_ at 806 and 770 nm for Ir[DMTPFPhl](py)_2_ and Au[DMTPFPhl], respectively (Figure 4). These absorption maxima are some 200 nm red-shifted relative to those observed for Ir^49−53^ and Au^54−58^ corroles, respectively, indicating a significantly smaller HOMO–LUMO gap in the case of the metallophlorins. The same picture also emerges from cyclic voltammetry measurements, as described below.

**Figure 5:**
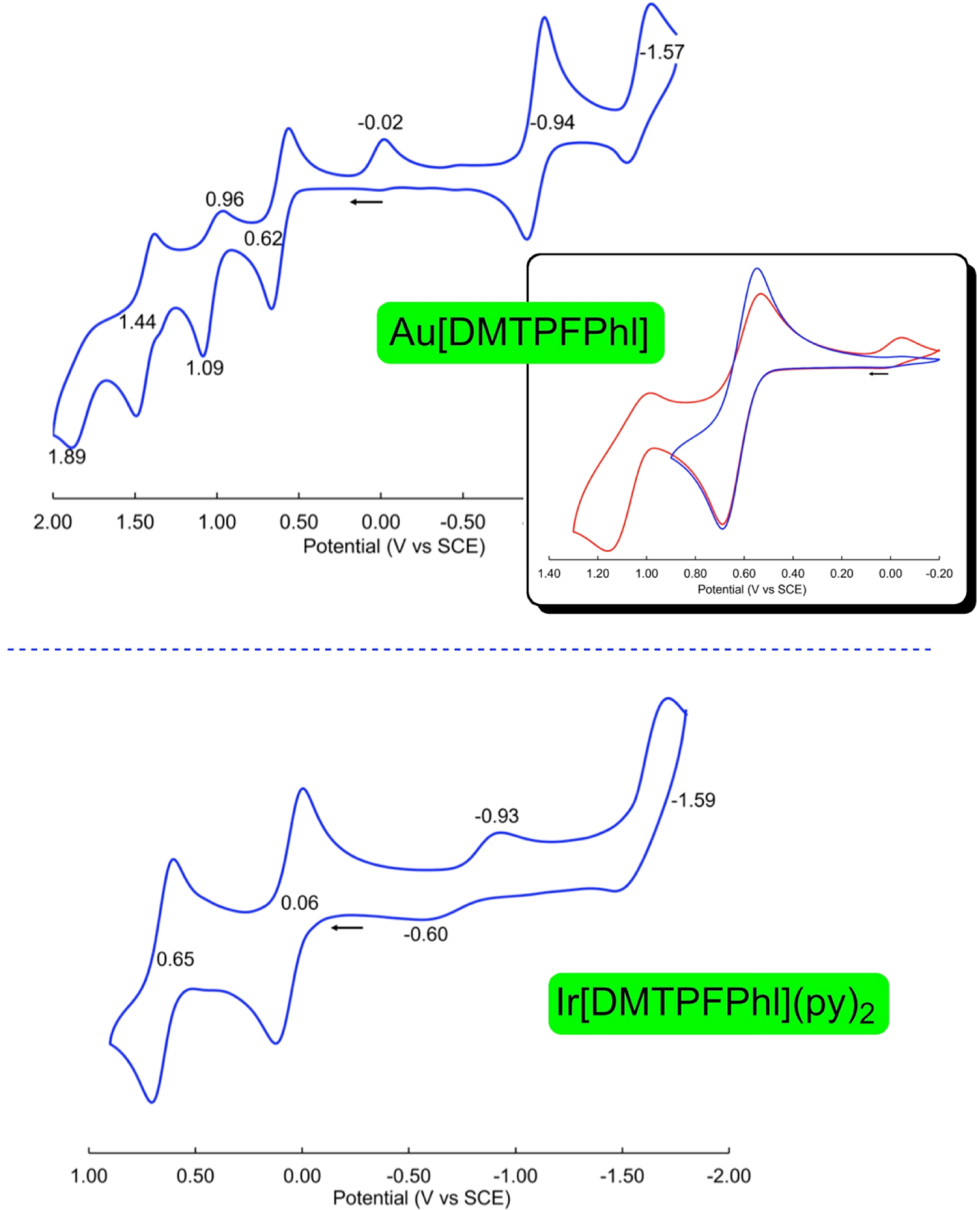
Cyclic voltammograms in dry dichloromethane with 0.1 M TBAPF_6_ at ambient temperature. Scan rate = 100 mV/s. The inset of Au[DMTPFPhl] shows the first oxidation and the first and second oxidation as separate scans.

Au[DMTPFPhl] exhibits four oxidations, of which the first and third are essentially reversible and the second and fourth are partially irreversible.^28^ Following oxidation over the full positive potential range, the reduction sweep exhibits a certain hysteresis in the form of a peak at −0.02 V vs saturated calomel electrode (SCE). Performing separate scans for the first oxidation and the first and second oxidation reveals that the peak at −0.02 V vs SCE is due to the quasi-reversibility of the second oxidation. The complex, however, reaches its normal neutral state before reduction to the anion commences. The first oxidation and reduction potential of Au[DMTPFPhl] at 0.62 and −0.94 V vs SCE, respectively, translate to a rather low electrochemical HOMO–LUMO gap of 1.56 V. A similarly low electrochemical HOMO–LUMO gap is also observed for Ir[DMTPFPhl](py)_2_ (Figure 5). These HOMO–LUMO gaps are well over half a volt lower than those observed for Ir and Au corroles.

As expected on the basis DFT calculations described above (Table 1), the redox potentials of the two metallophlorins examined differ substantially: both the first oxidation and reduction potentials of Ir[DMTPFPhl](py)_2_ are shifted to more negative potentials by some 600 mV relative to those of Au[DMTPFPhl], an indication that the phlorin macrocycle in the Ir complex is exceptionally electron-rich. For both metals, the redox-active MOs (the HOMO and the LUMO) are similar in character, except that, as discussed above, ruffling lifts the orthogonality between the phlorin’s π-LUMO and Au 5d_*x*^2^-*y*^2^_ orbital (Figures 1 and 2), so the reduction in the Au case occurs in a partially metal-centered manner. The electron-richness of Ir phlorins relative to their Au counterparts nicely mirrors the previously noted electron-richness of Ir corroles relative to Au corroles. Indeed, in the absence of strongly electron-withdrawing substituents, the reduction potentials of Ir corroles are generally not observable within the potential window of common electrochemical solvents.^52^ Finally, the origin of the small feature at −0.93 V for Ir[DMTPFPhl](py)_2_ remains uncertain, but a plausible explanation involves loss of one pyridine ligand and reduction of the resulting five-coordinate complex Ir[DMTPFPhl](py).

### NIR Phosphorescence of Phlorins

**Figure 6:**
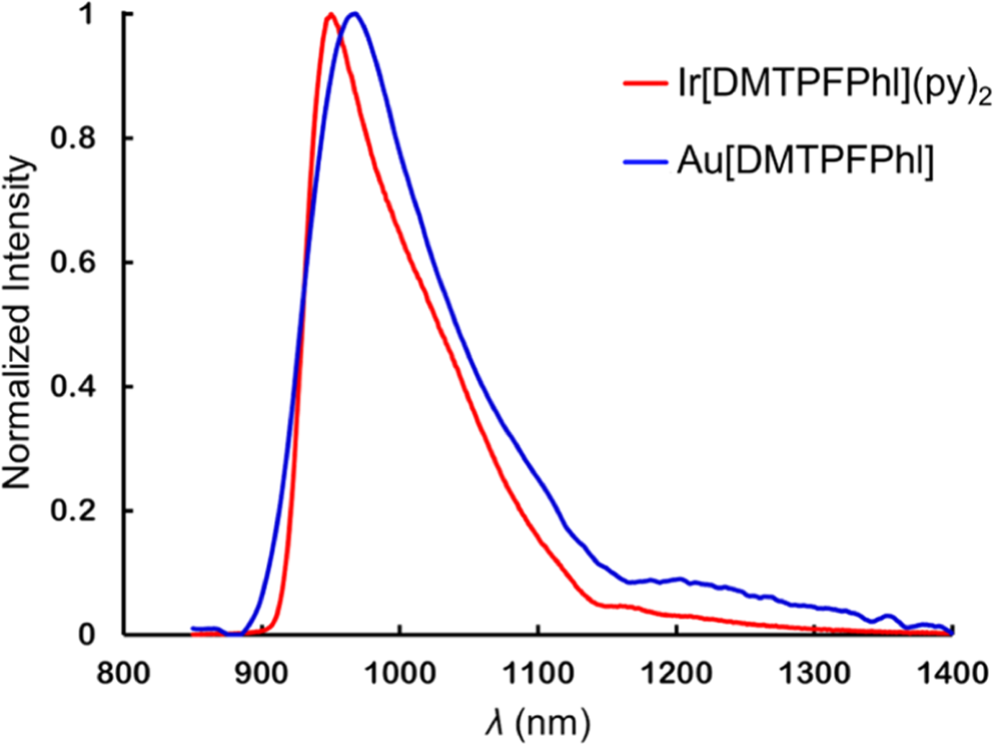
NIR phosphorescence spectra of Ir[DMTPFPhl](py)_2_ and Au[DMTPFPhl].

The phlorins exhibited weak near-infrared phosphorescence in the emission region of 850–1150 nm in anoxic toluene when excited with a 405 nm LED excitation source at room temperature. Ir[DMTPFPhl](py)_2_, with a phosphorescence quantum yield of 0.01%, was observed to exhibit a stronger phosphorescence relative to Au[DMTPFPhl], whose phosphorescence quantum yield was too low to be reliably determined by the relative method (Table 2). Although the overall phosphorescence profiles of both phlorins are similar, Au[DMTPFPhl] exhibits a red-shifted phosphorescence maximum (967 nm) compared to Ir[DMTPFPhl](py)_2_ (950 nm, Figure 6), emphasizing the importance of the transition metal center in determining the exact energetics of the luminescence. Their phosphorescence lifetimes, however, are similar: 22 μs for Au[DMTPFPhl] and 20 μs for Ir[DMTPFPhl](py)_2_ (see Figures S3 and S4).

**Table 2:** Summary of the Phosphorescence and Lifetime of the Phlorins

compounds	emission maxima (nm)	lifetime (μs)	singlet oxygen quantum yield (%)	phosphorescence quantum yield (%)	references
Ir[DMTPFPhl](py)_2_	950	20	40	0.01	this work
Au[DMTPFPhl]	967	22.3	28	a	this work
Au[TPFPC]	751	170	–b	0.68	(48)
Ir[T*p*CF_3_PC](py)_2_	836	5.6	71	0.04	(52)

a Too weak to be reliably determined by the relative method.

b “–” Not reported.

### Singlet Oxygen Quantum Yield

The singlet oxygen sensitization quantum yield was determined by a chemical method using 9,10-diphenylanthracene as the singlet oxygen acceptor and methylene blue as the reference dye (φ = 48%). Despite their weak near-infrared phosphorescence relative to 5d metallocorroles,^6,7,51,52,56,57,59,60^ both Au[DMTPFPhl] and Ir[DMTPFPhl](py)_2_ were found to sensitize singlet oxygen formation with moderate quantum yields of 28 and 40%, respectively. These singlet oxygen quantum yields are comparable to those exhibited by a number of free-base (34–86%)^61^ and 5d metallocorroles^6,7,62^ as well as transition metal isocorroles^9^ but are lower than those exhibited by Ga (51–77%),^63^ ReO^64,65^ (72%), and OsN corroles (76–95%).^66^Figure S10 depicts the degradation of the absorbance profile of the sensitizers in the presence of singlet oxygen. Their strong NIR absorption and moderate singlet oxygen-sensitizing ability encourage further examination of metallophlorins as sensitizers in photodynamic therapy.

## Conclusions

We have presented a combined theoretical and experimental study of iridium and gold phlorin derivatives that, in our view, has significantly deepened our understanding of key electronic structural characteristics of metallophlorins. DFT calculations, NIR absorption phosphorescence, and electrochemical measurements all indicate a substantially smaller HOMO–LUMO gap relative to typical, electronically innocent metalloporphyrins and metallocorroles. Interestingly, the Ir complex was found to exhibit dramatically lower redox potentials (by a margin of some 600 mV) relative to its Au counterpart, indicating a much more electron-rich macrocycle in the former. Both the Ir and Au complexes were found to photosensitize singlet oxygen formation, with quantum yields of 40 and 28%, respectively.

## Experimental Section

### Materials and Instruments

Reagents and solvents were used as purchased, unless noted otherwise. Pyridine was predried over and distilled from NaOH and stored over 4 Å molecular sieves. Dichloromethane was predried over and distilled from P_4_O_10_ and stored over activated 4 Å molecular sieves. 5,5-Dimethyl-10,15,20-tris(pentafluorophenyl)phlorin was prepared as previously described.^28^ Ultraviolet–visible (UV–vis) spectra were recorded on an HP 8454 spectrophotometer in CH_2_Cl_2_. ^1^H (400 MHz) and ^19^F (376 MHz) NMR spectra were acquired on a 400 MHz Bruker Avance III HD spectrometer equipped with a 5 mm SmartProbe BB/1H (BB = ^19^F, ^31^P–^15^N) in CDCl_3_ (δ = 7.26 ppm) and 2,2,2-trifluoroethanol-*d*_3_ (TFE-*d*_3_, ^19^F δ = −77.8 ppm), respectively. High-resolution electrospray ionization mass spectra (HR-ESI-MS) were recorded on an LTQ Orbitrap XL spectrometer.

Cyclic voltammetry was carried out at ambient temperature with a Gamry Reference 620 potentiostat equipped with a three-electrode system: a 3 mm disk glassy carbon working electrode, a platinum wire counter electrode, and a saturated calomel reference electrode (SCE). Tetra(*n*-butyl)ammonium hexafluorophosphate was used as the supporting electrolyte. Anhydrous CH_2_Cl_2_ (Aldrich) was used as the solvent. The electrolyte solution was purged with argon for at least 2 min prior to all measurements, which were carried out under an argon blanket. The glassy carbon working electrode was polished using a polishing pad and 0.05 μm polishing alumina from ALS, Japan. All potentials were referenced to the SCE.

### Synthesis of Gold 5,5-Dimethyl-10,15,20-tris(pentafluorophenyl)phlorin

Into a Schlenk tube charged with free-base phlorin (24 mg, 0.0286 mmol) and gold(III) acetate (5 equiv, 54 mg, 0.144 mmol) and preflushed with argon, dry pyridine (4 mL) was introduced under argon flow. After 5 min of degassing, the mixture was stirred at 50 °C for 50 min, at which point TLC (with 7:3 *n*-hexane/CH_2_Cl_2_ as eluent) on neutral alumina indicated that the free-base phlorin had been completely consumed. The suspension obtained was filtered through Celite and the filtrate was evaporated. The residue obtained was dissolved in a minimum volume of CH_2_Cl_2_ and placed on a silica gel column (18 cm × 3 cm). Eluting with 17:3 hexane/CH_2_Cl_2_ yielded a yellowish-brown band identified as the desired gold(III) phlorin (12 mg, 0.0116 mmol, 41%). UV–vis λ_max_ (nm) [ε × 10^–4^, (M^–1^·cm^–1^)]: 298 (1.96), 393 (2.19), 449 (5.72), 539 (0.32), 770 (1.39). ^1^H NMR (CDCl_3_, δ = 7.26 ppm): δ 7.39 (d, 2H, *J* = 5.2 Hz, β-H); 7.34 (d, 2H, *J* = 4.12 Hz, β-H), 7.21–7.15 (overlapping d, 4H, β-H); 1.53 (br s, 6H; 5,5-Me). ^19^F NMR: δ −137.83 (dd, 2F, *J* = 6.5 and 2.3 Hz, 15-*o*), −138.48 (dd, 4F, *J* = 5.96 and 1.74 Hz, 10, 20-*o*), −152.85 (t, 2F, *J* = 5.57 Hz, 10, 20-*p*), −153.10 (t, 1F, *J* = 5.57 Hz, 15-*p*), −161.51 (tm, 6F, *J* = 5.57 Hz, 10,15, 20-*m*). HRMS (ESI^+^, major isotopomer): [M]^+^ = 1032.0669 (expt), 1032.0650 (calcd).

### Synthesis of Iridium-bispyridine 5,5-Dimethyl-10,15,20-tris(pentafluorophenyl)phlorin

A round-bottom flask was charged with free-base phlorin (26 mg, 0.031 mmol), [Ir(cod)Cl]_2_ (20 equiv, 416 mg, 0.62 mmol), and K_2_CO_3_ (163 equiv, 0.7 g, 5.06 mmol). Upon flushing with argon, dry THF (10 mL) was introduced under argon flow. After 5 min of degassing, the green suspension was stirred at reflux for 1.5 h, at which point TLC (with 1:1 *n*-hexane/CH_2_Cl_2_) on neutral alumina indicated the continued presence of free-base phlorin. An additional quantity of K_2_CO_3_ (226 equiv, 0.97 g, 7.02 mmol) was added to the reaction mixture, and refluxing was continued for an additional 2.5 h. By that time, the reaction mixture turned brown, indicating that the free-base phlorin had been consumed. Heating was discontinued, and pyridine (1 mL) was added to the reaction mixture while stirring was continued. After 0.5 h, the mixture was filtered through a pad of Celite, and the filtrate was evaporated. The residue obtained was dissolved in a minimum volume of CH_2_Cl_2_ and chromatographed on a basic alumina column (7 cm × 2 cm) with CH_2_Cl_2_ as eluent, whereupon the desired complex was obtained as a brown solid (14 mg, 0.0118 mmol, 38.1%) after washing with *n*-pentane. UV–vis λ_max_ (nm) [ε × 10^–4^, (M^–1^·cm^–1^)]: 310 (1.53), 447 (4.52), 806 (1.22). ^1^H NMR (CDCl_3_, δ = 7.26 ppm): δ 7.18 (t, 2H, *J* = 7.6 Hz, *p-*Py); 7.01 (d, 2H, *J* = 4.0 Hz, β-H), 6.99 (d, 2H, *J* = 4.96 Hz β-H); 6.79 (d, 2H, *J* = 4.64 Hz, β-H), 6.69 (br d, 2H, β-H), 6.46 (t, 4H, *J* = 6.8 Hz, *m-*Py), 6.03 (d, 4H, *J* = 5.4 Hz, *o-*Py), 1.20 (s, 6H; 5,5-Me). ^19^F NMR: δ – 139.80 (dd, 2F, *J* = 6.76 and 2.3 Hz, 15-*o*), – 140.60 (dd, 4F, *J* = 6.52 and 2.21 Hz, 10, 20-*o*), – 155.50 (t, 2F, *J* = 5.55 Hz, 10, 20-*p*), – 155.94 (t, 1F, *J* = 5.57 Hz, 15-*p*), – 162.81 to −163.51 (overlapping tm, 6F, 10,15,20-*m*). HRMS (ESI^+^, major isotopomer): [M]^+^ = 1186.1432 (expt), 1186.1450 (calcd).

### X-Ray Diffraction Analysis

Crystallographic quality crystals of Au[DMTPFPhl] were obtained by diffusion of water vapor into a concentrated solution of the complex in pyridine. A suitable crystal with dimensions 0.38 × 0.10 × 0.08 mm^3^ was mounted on MiTeGen holder oil on a Nonius Kappa Apex II diffractometer. The crystal was kept at a steady *T* = 110.0(1) K during data collection. The structure was solved with ShelXT^67^ using dual methods and Olex2 1.5 as the graphical interface.^68^ The model was refined with ShelXL^69^ 2018/3 using full-matrix least-squares minimization on *F*^2^ using the ShelXle GUI. All non-hydrogen atoms were refined anisotropically. Hydrogen atom positions were calculated geometrically and refined using the riding model.

### Selected Crystal Data for Au[DMTPFPhl]

Chemical formula C_45_H_19_AuF_15_N_5_, *M*_r_ = 1111.62, monoclinic, *P*2_1_/*c* (No. 14), *a* = 25.4240(8) Å, *b* = 15.4865(6) Å, *c* = 9.8650(4) Å, *b* = 99.817(2)°, *a* = *g* = 90°, *V* = 3827.3(2) Å^3^, *T* = 110.0(1) K, *Z* = 4, *Z*′ = 1, *m*(Mo·Kα_1_) = 3.961, 133871 reflections measured, 8786 unique (*R*_int_ = 0.1257) which were used in all calculations. The final *w*R**_2_ was 0.0629 (all data) and *R*_1_ was 0.0329 (I ≥ 2 *s*(I)).

### Photophysical Measurements

All photophysical studies (phosphorescence and lifetime) were performed in sealed cuvettes under dry N_2_ using degassed toluene. NIR phosphorescence and lifetimes were measured on an OLIS NIR CPL Solo spectrofluorometer using ∼10^–4^ M toluene solutions and a 405 nm LED light source. Lifetime spectra were collected using pulsed excitation at 405 nm and time-resolved emission measurements fixed at the peak of the strongest emission. A first-order exponential decay curve was fitted to the collected data to estimate the fluorescence lifetime (τ_obs_). Values are reported as measured lifetimes (observation wavelength). The NIR PMT detector is a HAMAMATSU model H10330C-75-C2 cooled at −60 °C using an internal air-cooled thermoelectric cooler. The detector uses an InGaAs photocathode material. The sample is illuminated with LEDs (Everlight) driven to an output of 1000 mW.

For phosphorescence quantum yields, the dye solution in a screw-capped cuvette was unscrewed prior to each measurement, diluted to a new concentration, and then purged with nitrogen for 3 min. Yb(tta)_3_(H_2_O)_2_ was used as the reference compound (QY = 0.35% in toluene). Singlet oxygen quantum yields were calculated by the relative method using 9,10-diphenylanthracene as the singlet oxygen acceptor. The metallophlorins were dissolved in 9:1 v/v EtOH/THF to a concentration of approximately 15 μM, adjusted for identical absorption at the excitation wavelength. A 0.28 mM solution of 9,10-diphenylanthracene in 9:1 v/v EtOH/THF was also prepared. Equal volumes of the 9,10-diphenylanthracene solution and each photosensitizer solution were then mixed in a 1 cm path-length cuvette. The mixture was saturated with oxygen by bubbling air through it for 3 min. The cuvette was sealed and the absorbance was measured before it was irradiated with light (two 405 nm Everlight LEDs each driven to an output of 1000 mW, 10 nm slit) for 3 min. The cuvette was then shaken and an absorbance spectrum was remeasured. The experiment was repeated 4 times. Singlet oxygen quantum yields were calculated from the slope of the curve (absorbance at 372 nm vs. time) using methylene blue as the reference (φ = 0.48).

### Computational Methods

The DFT calculations described above employed the scalar-relativistic ZORA^70−72^ (zeroth-order regular approximation to the Dirac equation) Hamiltonian, the dispersion-corrected B3LYP-D3 method, and all-electron ZORA STO/TZ2P basis sets, all as implemented in the ADF program system.^73,74^ A number of additional exchange–correlation functionals were also examined, but both the optimized geometries and calculated IPs showed minimal variations across the different methods;^39−43^ accordingly, only the B3LYP-D3 results have been reported here. The following point group symmetries were used: Pt[Por] (*D*_4h_), Au[Cor] (*C*_2v_), Au[Phl] (*C*_s_), and Ir[Phl](NH_3_)_2_ (*C*_s_). All energies were calculated in the gas phase with the Δ*S*CF method, i.e., as differences in total electronic energy between initial and final states. When needed, different ionized and excited states were calculated by specifying the number of electrons in each irrep; these occupations are all explicitly specified in the Supporting Information. Note also that the optimized geometry of {Pt[Por]}^+^ (used in the calculation of the adiabatic IP of the neutral compound) is only *C*_4h_, as a result of skeletal bond length alternations arising from a pseudo-Jahn–Teller distortion.^75^

## Data Availability

All data generated or analyzed in this study are included in this published article and its Supporting Information.

## Supporting Information Available

ESI mass spectra; selected photophysical data; and optimized DFT coordinates (PDF)
