# Supplementary material for: Electronic Structure of Metallophlorins: Lessons from Iridium and Gold Phlorin Derivatives
Source: Inorg Chem. 2024 May 14;63(21):9842–53. doi: 10.1021/acs.inorgchem.4c00483 (PMC11134504; doi:10.1021/acs.inorgchem.4c00483)
Supplement: Supplementary file 1 — ic4c00483_si_001.pdf [file ic4c00483_si_001.pdf]

Supporting Information

Electronic Structure of Metallophlorins: Lessons from Iridium and Gold Phlorin Derivatives

Simon Larsen,[a] Joseph A. Adewuyi,[b] Kolle E. Thomas,[a] Jeanet Conradie,[a,c]

Yoann Rousselin,[d] Gaël Ung,*[,b] and Abhik Ghosh*[,a]

[a]Department of Chemistry, University of Tromsø, N-9037 Tromsø, Norway

[b]Department of Chemistry, University of Connecticut, 55 N. Eagleville Rd, Storrs, CT 06269, USA

[c]Department of Chemistry, University of the Free State, P.O. Box 339, Bloemfontein 9300, Republic of South Africa.

[d]ICMUB, UMR CNRS 6302, Université Bourgogne Franche-Comte, BP 47870, 21078 Dijon Cedex, France

* Abhik Ghosh: abhik.ghosh@uit.no; Gaël Ung: gael.ung@uconn.edu

Contents **Page**

A. ^{1}H NMR spectra 2

B. ESI-MS spectra (positive mode) 4

C. Phosphorescence lifetime measurements 5

D. Singlet oxygen sensitization measurements 6

E. Degradation profile 7

F. Optimized Cartesian coordinates (Å) 8

A. ^{1}H NMR spectra

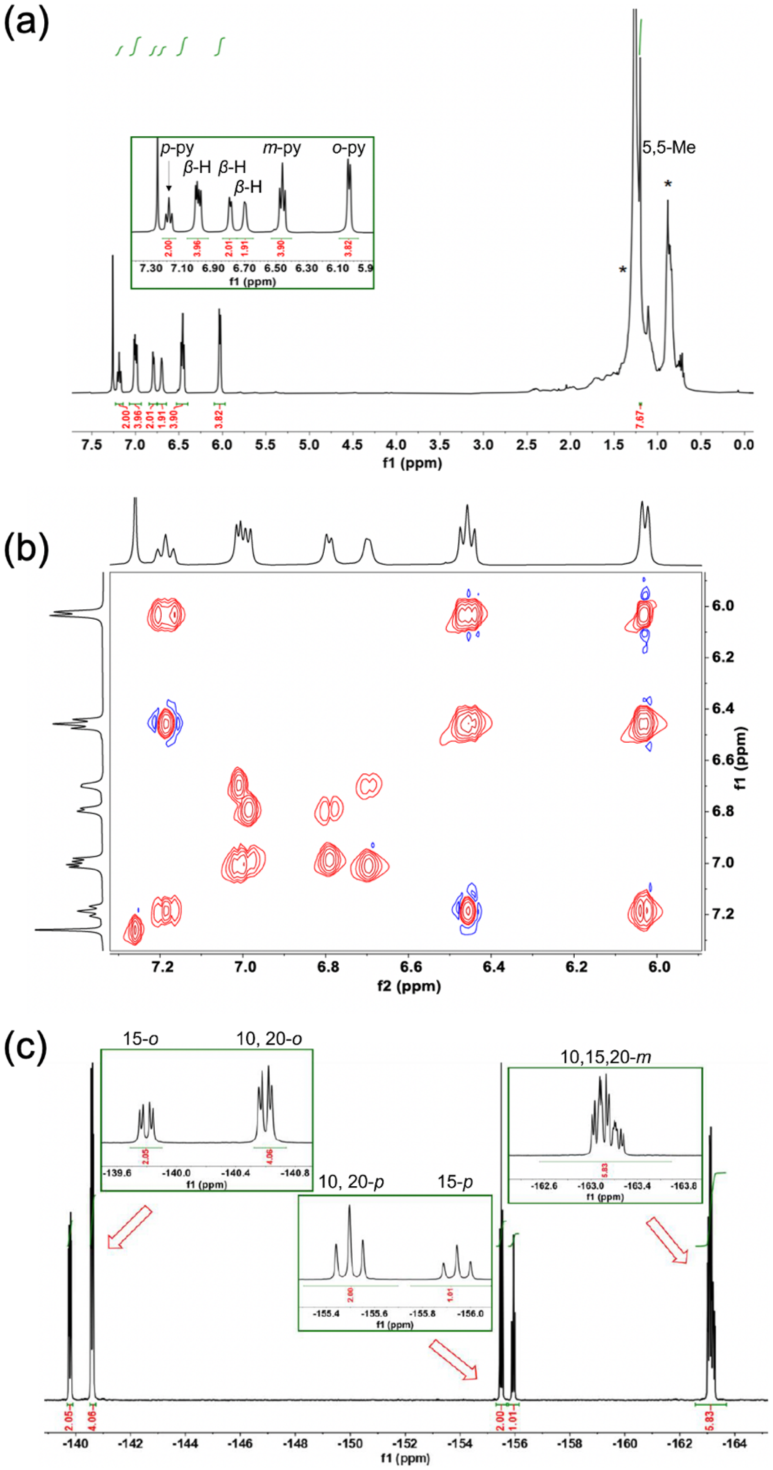

Figure S1. Selected NMR spectra for Ir[DMTPFPhl](py)$_2$ in $CDCl_3$ at ambient temperature: (a) ^{1}H NMR, (b) ^{1}H-^{1}H TOCSY, and (c) ^{19}F NMR spectra.

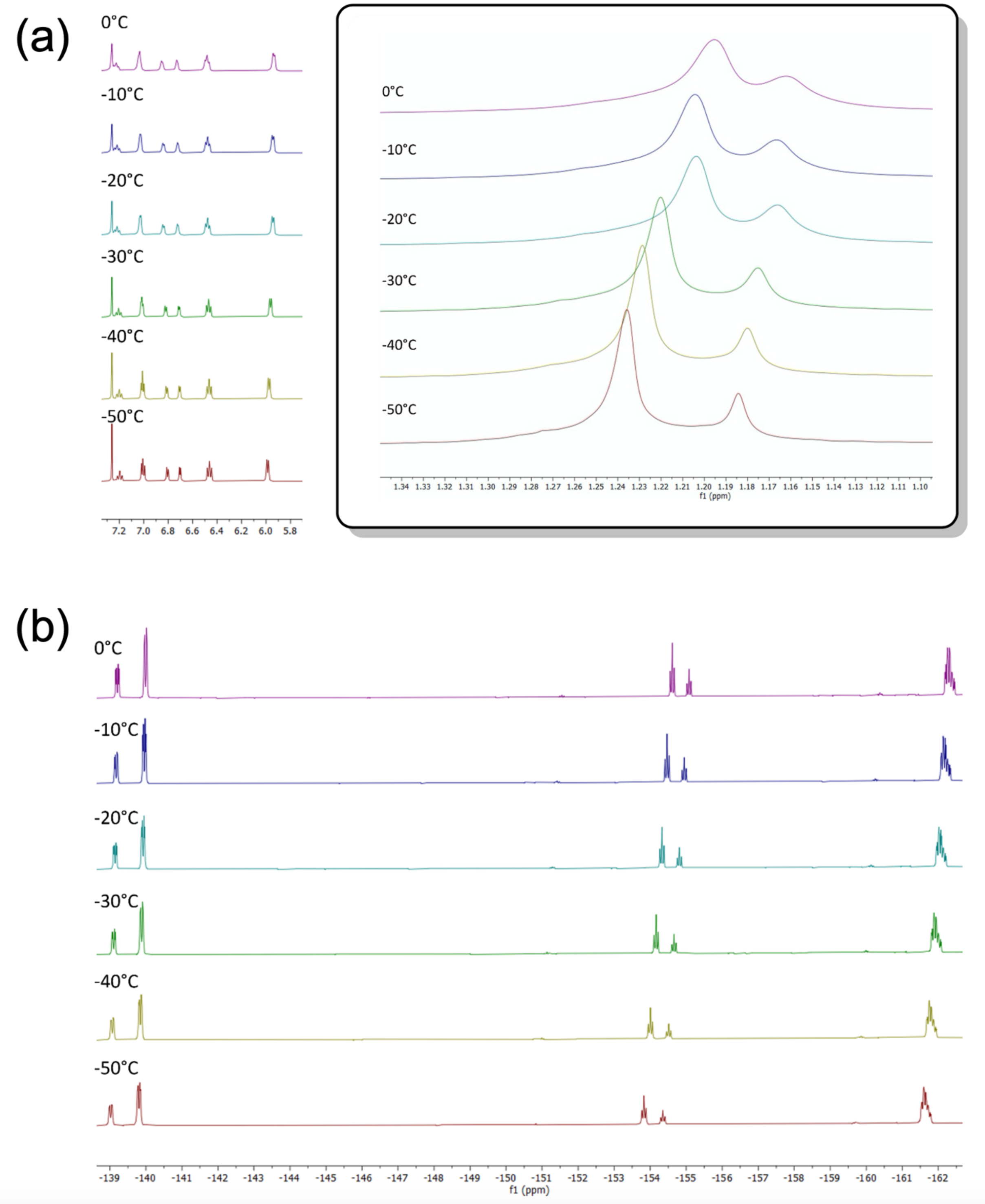

Figure S2. Temperature-dependent (a) ^{1}H and (b) ^{19}F NMR spectra of Ir[DMTPFPhl](py)$_2$ in $CDCl_3$ from 0°C to -50 °C. Inset: The taller peak to the left is from residual pentane and the smaller peak to the right corresponds to the 5,5-dimethyl protons.

B. ESI-MS spectra (positive mode)

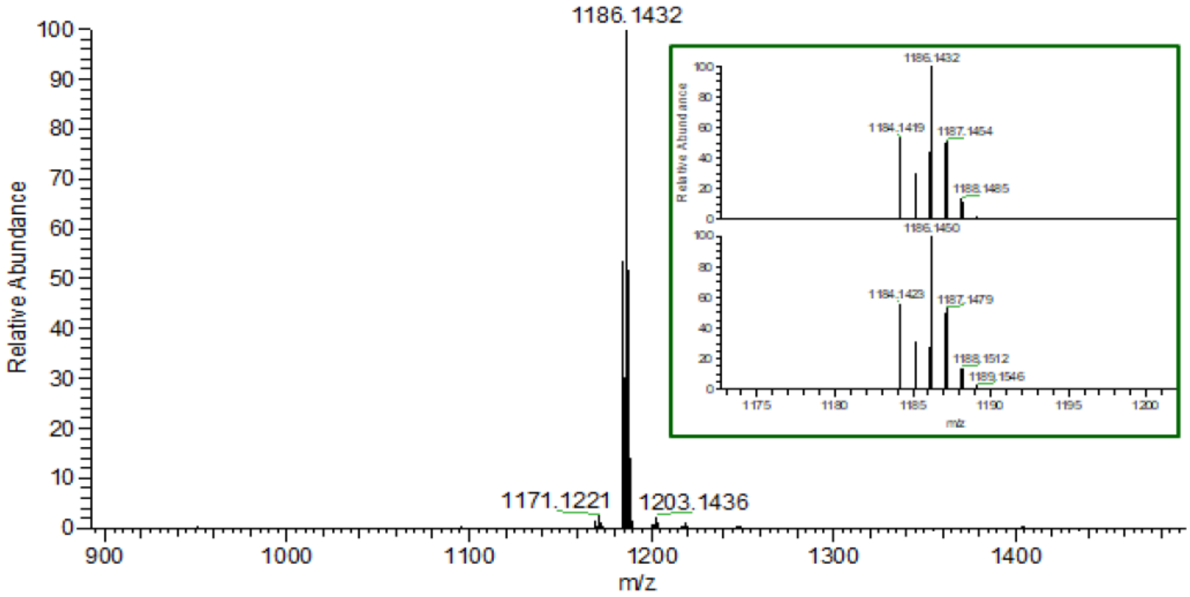

Figure S3. ESI-MS spectrum of Ir[DMTPFPhl](py)$_2$.

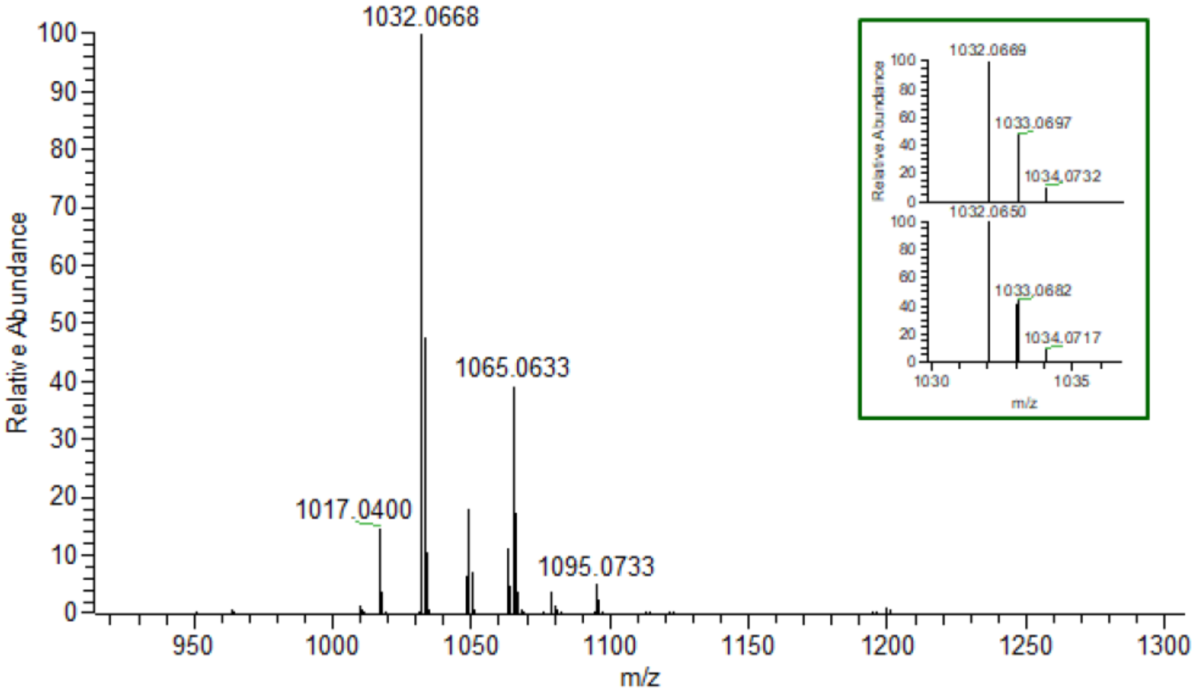

Figure S4. ESI-MS spectrum of Au[DMTPFPhl].

C. Phosphorescence lifetime measurements

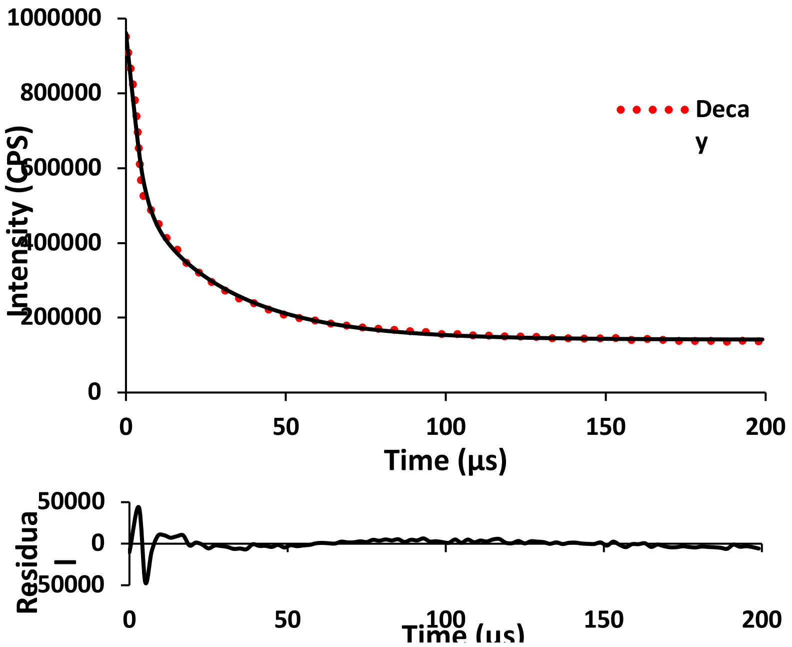

Figure S5. Lifetime of Ir[DMTPFPhl](py)$_2$ at 3.53 x 10^{-4} M fitted to a biexponential function. Average of 50 scans. Lifetimes = 43.3 µs and 4.5 µs at 953 nm.

Note: While the lifetime plot of Au[DMTPFPhl] fits to a single exponential function (Figure S4) with a lifetime value of 22.3 µs, the lifetime plot of Ir[DMTPFPhl](py)$_2$ could only be fit to a biexponential function with lifetime values corresponding to 43.3 µs and 4.5 µs. The longer lifetime value of 43.3 µs could be attributed to Ir[DMTPFPhl](py)$_2$ while the shorter lifetime value of 4.5 µs may be associated to an impurity from the photodegradation of the Ir phlorin from the pulsed excitation of light during lifetime measurement. The low lifetime value also suggests that the degradation impurity is *not* an iridium complex.

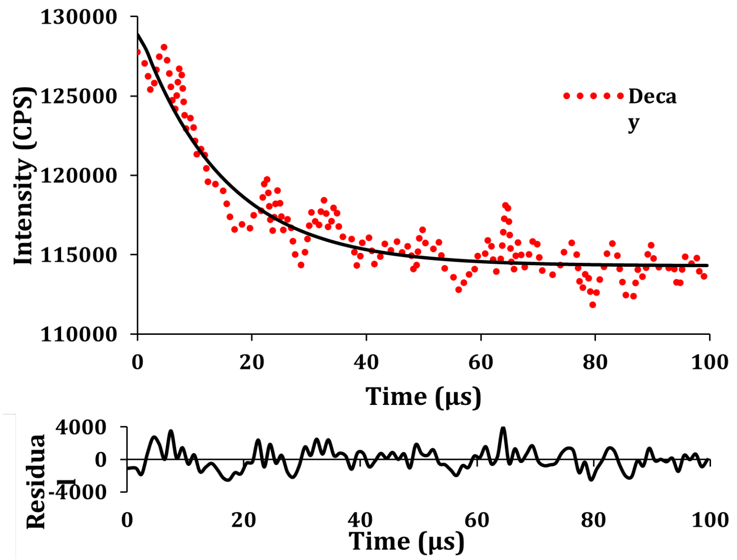

Figure S6. Lifetime of Au[DMTPFPhl] at 3.58 x 10-4 M. Average of 50 scans. Lifetime = 22.3 µs (970 nm)

D. Singlet oxygen sensitization measurements

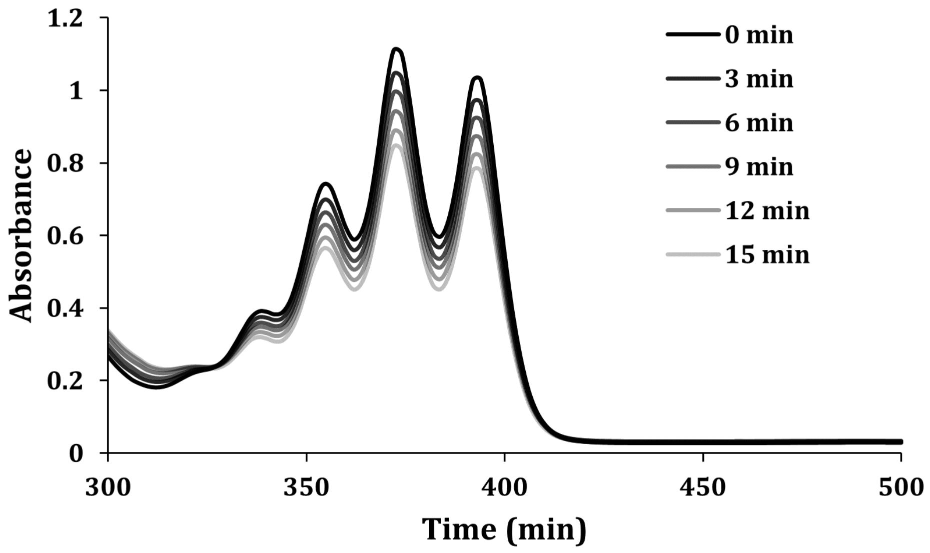

Figure S7. Absorbance spectra showing degradation of 9,10-diphenylanthracene (0.28 mM) in an air-saturated solution (9:1 EtOH/THF) in the presence of Methylene Blue (15 μM) upon irradiation with 405 nm LED.

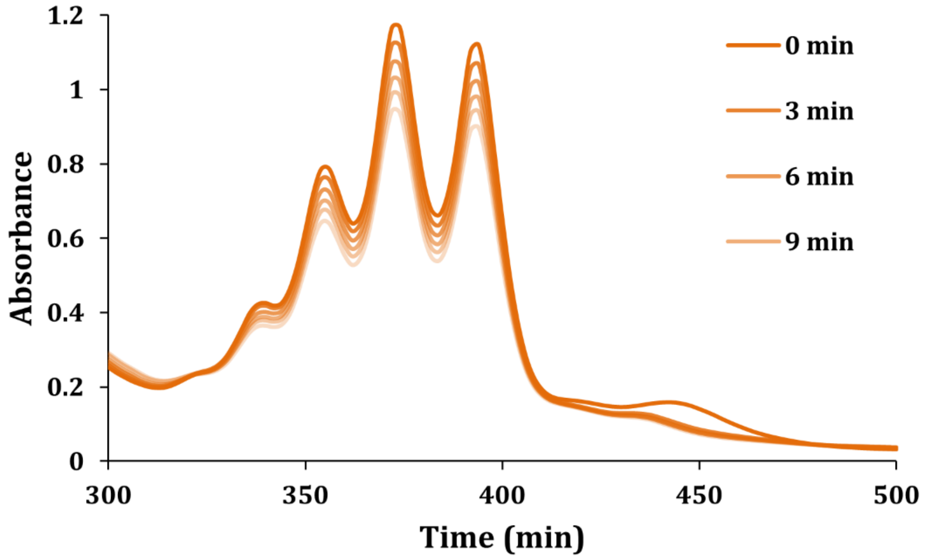

Figure S8. Absorbance spectra showing degradation of 9,10-diphenylanthracene (0.28 mM) in an air-saturated solution (9:1 EtOH/THF) in the presence of Ir[DMTPFPhl]$(py)_2$ (15 μM) upon irradiation with 405 nm LED.

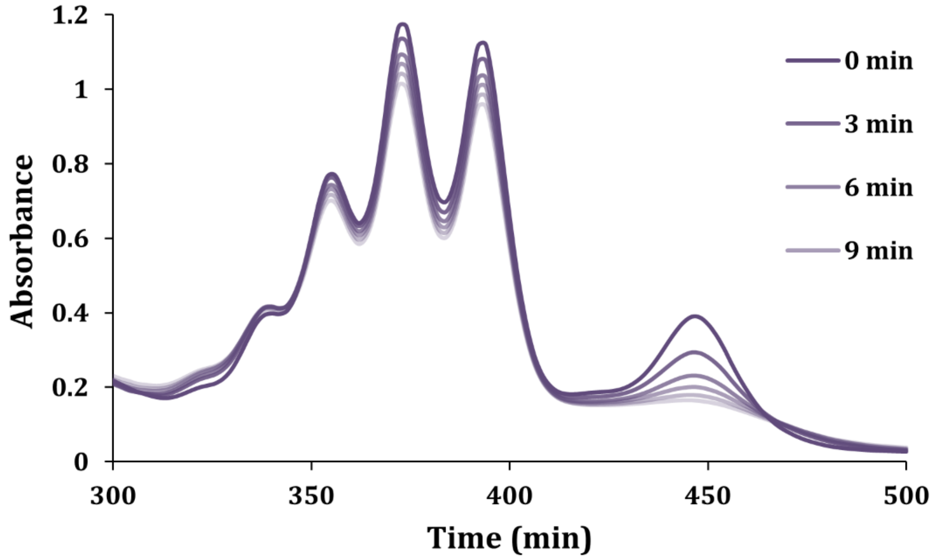

Figure S9. Absorbance spectra showing degradation of 9,10-diphenylanthracene (0.28 mM) in an air-saturated solution (9:1 EtOH/THF) in the presence of Au[DMTPFPhl] (15 µM) upon irradiation with 405 nm LED.

E. Degradation profile

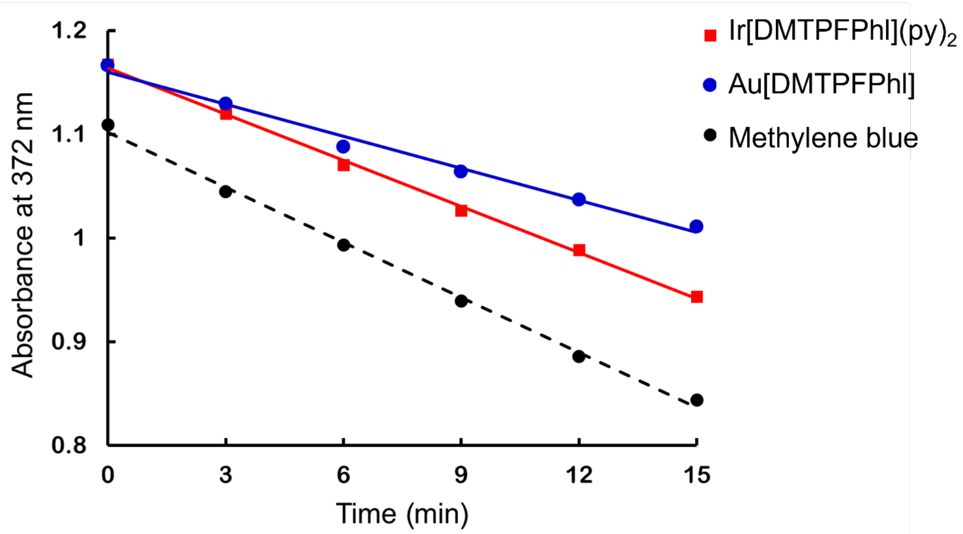

Figure S10. Degradation profile of 9,10-diphenylanthracene (0.28 mM) in an air-saturated solution (9:1 EtOH/THF) in the presence of metallophlorins (15 µM) upon irradiation with 405 nm LED.

F. Optimized Cartesian coordinates (Å)

All compounds were optimized the ZORA Hamiltonian, all-electron ZORA/TZ2P basis sets, and the B3LYP functional augmented with Grimme's D3 dispersion correction.

1. Au[Phl] (ground state), C_s; all-electron occupation (irrep α|β): a′ 70|70, a″ 50|50

Au	0.000508000	0.076265000	0.000000000
C	1.206377000	-0.468263000	2.751121000
C	1.206377000	-0.468263000	-2.751121000
C	2.453778000	-0.691983000	3.350089000
C	2.453778000	-0.691983000	-3.350089000
C	2.772530000	0.005060000	1.241758000
C	2.772530000	0.005060000	-1.241758000
C	3.361089000	0.576385000	0.000000000
C	3.430108000	-0.400476000	2.401109000
C	3.430108000	-0.400476000	-2.401109000
C	-0.050768000	-0.478212000	3.367956000
C	-0.050768000	-0.478212000	-3.367956000
C	-1.239077000	-0.140859000	2.781803000
C	-1.239077000	-0.140859000	-2.781803000
C	-2.505968000	0.000013000	3.432172000
C	-2.505968000	0.000013000	-3.432172000
C	-2.752957000	0.427667000	1.227935000
C	-2.752957000	0.427667000	-1.227935000
C	-3.365436000	0.625563000	0.000000000
C	-3.420506000	0.367986000	2.498828000
C	-3.420506000	0.367986000	-2.498828000
H	2.598200000	-0.993816000	4.374378000
H	2.598200000	-0.993816000	-4.374378000
H	3.210128000	1.665417000	0.000000000
H	4.436302000	0.408091000	0.000000000
H	4.499728000	-0.432959000	2.526062000
H	4.499728000	-0.432959000	-2.526062000
H	-0.073457000	-0.723302000	4.420710000
H	-0.073457000	-0.723302000	-4.420710000
H	-2.663669000	-0.161007000	4.486331000
H	-2.663669000	-0.161007000	-4.486331000
H	-4.424790000	0.833212000	0.000000000
H	-4.472033000	0.558072000	2.636866000
H	-4.472033000	0.558072000	-2.636866000
N	1.430450000	-0.056670000	1.440458000
N	1.430450000	-0.056670000	-1.440458000
N	-1.429009000	0.147032000	1.431260000
N	-1.429009000	0.147032000	-1.431260000

2. Au[Phl] (anion), C_s; all-electron occupation (irrep α|β): a′ 70|70, a″ 51|50

Au	0.014751000	0.157582000	0.000000000
C	1.243094000	-0.245752000	2.837418000
C	1.243094000	-0.245752000	-2.837418000
C	2.468328000	-0.601548000	3.442044000
C	2.468328000	-0.601548000	-3.442044000
C	2.823699000	-0.053622000	1.286933000
C	2.823699000	-0.053622000	-1.286933000
C	3.455084000	-0.498051000	2.459370000
C	3.455084000	-0.498051000	-2.459370000
C	3.464472000	0.392688000	0.000000000
C	-0.037143000	-0.214071000	3.435447000
C	-0.037143000	-0.214071000	-3.435447000
C	-1.266929000	-0.020523000	2.838893000
C	-1.266929000	-0.020523000	-2.838893000
C	-2.543244000	0.079867000	3.498732000
C	-2.543244000	0.079867000	-3.498732000
C	-2.826054000	0.256831000	1.254700000
C	-2.826054000	0.256831000	-1.254700000
C	-3.442994000	0.343672000	0.000000000
C	-3.492726000	0.264770000	2.538813000
C	-3.492726000	0.264770000	-2.538813000
H	2.591208000	-0.916110000	4.467107000
H	2.591208000	-0.916110000	-4.467107000
H	3.486302000	1.492532000	0.000000000
H	4.505271000	0.066193000	0.000000000
H	4.512157000	-0.686483000	2.570594000
H	4.512157000	-0.686483000	-2.570594000
H	-0.052925000	-0.367821000	4.508054000
H	-0.052925000	-0.367821000	-4.508054000
H	-2.687787000	0.032617000	4.567506000
H	-2.687787000	0.032617000	-4.567506000
H	-4.517863000	0.466978000	0.000000000
H	-4.557852000	0.371840000	2.677346000
H	-4.557852000	0.371840000	-2.677346000
N	1.504746000	0.095925000	1.519972000
N	1.504746000	0.095925000	-1.519972000
N	-1.495997000	0.093060000	1.480677000
N	-1.495997000	0.093060000	-1.480677000

3. Au[Phl] (cation), C_s; all-electron occupation (irrep α|β): a′ 70|69, a″ 50|50

Au	-0.007256000	0.012898000	0.000000000
C	1.192041000	-0.396056000	2.787006000
C	1.192041000	-0.396056000	-2.787006000
C	2.446883000	-0.547952000	3.426776000

C	2.446883000	-0.547952000	-3.426776000
C	2.765042000	-0.065062000	1.263820000
C	2.765042000	-0.065062000	-1.263820000
C	3.419825000	-0.336665000	2.480034000
C	3.419825000	-0.336665000	-2.480034000
C	3.429524000	0.334612000	0.000000000
C	-0.046590000	-0.396092000	3.393883000
C	-0.046590000	-0.396092000	-3.393883000
C	-1.256306000	-0.119534000	2.782385000
C	-1.256306000	-0.119534000	-2.782385000
C	-2.513597000	0.021117000	3.441059000
C	-2.513597000	0.021117000	-3.441059000
C	-2.775420000	0.360187000	1.231333000
C	-2.775420000	0.360187000	-1.231333000
C	-3.389673000	0.528582000	0.000000000
C	-3.441004000	0.333871000	2.495484000
C	-3.441004000	0.333871000	-2.495484000
H	2.576586000	-0.767296000	4.473553000
H	2.576586000	-0.767296000	-4.473553000
H	3.518500000	1.432449000	0.000000000
H	4.452162000	-0.039824000	0.000000000
H	4.488332000	-0.354085000	2.613893000
H	4.488332000	-0.354085000	-2.613893000
H	-0.069403000	-0.582416000	4.458473000
H	-0.069403000	-0.582416000	-4.458473000
H	-2.660942000	-0.098576000	4.501670000
H	-2.660942000	-0.098576000	-4.501670000
H	-4.450538000	0.730639000	0.000000000
H	-4.494483000	0.513421000	2.630631000
H	-4.494483000	0.513421000	-2.630631000
N	1.428016000	-0.112804000	1.440792000
N	1.428016000	-0.112804000	-1.440792000
N	-1.442712000	0.099016000	1.433099000
N	-1.442712000	0.099016000	-1.433099000

4. Au[Phl] (triplet), C_s; all-electron occupation (irrep $\alpha|\beta$): a′ 70|69, a″ 51|50

Au	0.003352000	0.093078000	0.000000000
C	1.230680000	-0.190635000	2.854032000
C	1.230680000	-0.190635000	-2.854032000
C	2.472820000	-0.461537000	3.502650000
C	2.472820000	-0.461537000	-3.502650000
C	2.811264000	-0.115671000	1.306220000
C	2.811264000	-0.115671000	-1.306220000
C	3.453149000	-0.423338000	2.536784000
C	3.453149000	-0.423338000	-2.536784000
C	3.502428000	0.155878000	0.000000000
C	-0.033545000	-0.156112000	3.437233000
C	-0.033545000	-0.156112000	-3.437233000

C	-1.284538000	0.001038000	2.832897000
C	-1.284538000	0.001038000	-2.832897000
C	-2.555434000	0.092024000	3.499883000
C	-2.555434000	0.092024000	-3.499883000
C	-2.841428000	0.216529000	1.261330000
C	-2.841428000	0.216529000	-1.261330000
C	-3.449408000	0.282648000	0.000000000
C	-3.510772000	0.236815000	2.537530000
C	-3.510772000	0.236815000	-2.537530000
H	2.591493000	-0.675533000	4.552561000
H	2.591493000	-0.675533000	-4.552561000
H	3.769871000	1.223312000	0.000000000
H	4.454723000	-0.375748000	0.000000000
H	4.511196000	-0.582242000	2.668327000
H	4.511196000	-0.582242000	-2.668327000
H	-0.050940000	-0.276930000	4.513249000
H	-0.050940000	-0.276930000	-4.513249000
H	-2.696456000	0.063728000	4.568405000
H	-2.696456000	0.063728000	-4.568405000
H	-4.526071000	0.387682000	0.000000000
H	-4.575773000	0.331628000	2.675078000
H	-4.575773000	0.331628000	-2.675078000
N	1.499645000	0.027337000	1.509656000
N	1.499645000	0.027337000	-1.509656000
N	-1.506183000	0.080226000	1.486789000
N	-1.506183000	0.080226000	-1.486789000

5. Ir[Phl](NH_3)$_2$, C_s; all-electron occupation (irrep α|β): a′ 77|77, a″ 52|52

Ir	0.017169000	-0.092786000	0.000000000
C	1.205248000	0.358480000	2.804570000
C	1.205248000	0.358480000	-2.804570000
C	2.471010000	0.531316000	3.462043000
C	2.471010000	0.531316000	-3.462043000
C	2.796732000	0.248542000	1.241669000
C	2.796732000	0.248542000	-1.241669000
C	3.432755000	0.226046000	0.000000000
C	3.442803000	0.446030000	2.517149000
C	3.442803000	0.446030000	-2.517149000
C	-0.034711000	0.374825000	3.394130000
C	-0.034711000	0.374825000	-3.394130000
C	-1.276090000	0.106745000	2.786606000
C	-1.276090000	0.106745000	-2.786606000
C	-2.542560000	0.087895000	3.401160000
C	-2.542560000	0.087895000	-3.401160000
C	-2.737632000	-0.574288000	1.264429000
C	-2.737632000	-0.574288000	-1.264429000
C	-3.235591000	-1.197966000	0.000000000
C	-3.457175000	-0.335073000	2.441048000

C	-3.457175000	-0.335073000	-2.441048000
H	0.026858000	-2.588876000	0.825892000
H	0.026858000	-2.588876000	-0.825892000
H	0.499327000	2.490448000	0.000000000
H	1.426448000	-2.301979000	0.000000000
H	2.590497000	0.693443000	4.521436000
H	2.590497000	0.693443000	-4.521436000
H	4.508656000	0.533829000	2.652625000
H	4.508656000	0.533829000	-2.652625000
H	4.510831000	0.299094000	0.000000000
H	-0.061380000	0.583757000	4.455805000
H	-0.061380000	0.583757000	-4.455805000
H	-0.904587000	2.217380000	0.825959000
H	-0.904587000	2.217380000	-0.825959000
H	-2.734960000	0.331210000	4.433474000
H	-2.734960000	0.331210000	-4.433474000
H	-2.956309000	-2.264195000	0.000000000
H	-4.324832000	-1.176879000	0.000000000
H	-4.517078000	-0.486748000	2.565130000
H	-4.517078000	-0.486748000	-2.565130000
N	0.421302000	-2.147263000	0.000000000
N	1.449740000	0.171805000	1.447636000
N	1.449740000	0.171805000	-1.447636000
N	-0.368431000	1.962037000	0.000000000
N	-1.426293000	-0.293310000	1.460787000
N	-1.426293000	-0.293310000	-1.460787000

6. Ir[Phl]$(NH_3)_2$, C_{2v}; all-electron occupation (irrep $\alpha|\beta$): a_1 56|56, a_2 11|11, b_1 21|21 b_2 41|41

Ir	0.000000000	0.000000000	0.006937000
C	0.000000000	0.000000000	3.468801000
C	0.000000000	0.000000000	-3.556760000
C	0.000000000	1.238177000	2.833474000
C	0.000000000	1.298767000	-2.810644000
C	0.000000000	2.516878000	3.503475000
C	0.000000000	2.547463000	-3.445889000
C	0.000000000	2.824534000	1.271451000
C	0.000000000	2.838064000	-1.221311000
C	0.000000000	3.437712000	0.049728000
C	0.000000000	3.479879000	2.548523000
C	0.000000000	3.514702000	-2.451569000
C	0.000000000	-1.238177000	2.833474000
C	0.000000000	-1.298767000	-2.810644000
C	0.000000000	-2.516878000	3.503475000
C	0.000000000	-2.547463000	-3.445889000
C	0.000000000	-2.824534000	1.271451000
C	0.000000000	-2.838064000	-1.221311000
C	0.000000000	-3.437712000	0.049728000
C	0.000000000	-3.479879000	2.548523000

C	0.000000000	-3.514702000	-2.451569000
H	0.000000000	0.000000000	4.549477000
H	0.000000000	2.641390000	4.574323000
H	0.000000000	2.701141000	-4.513365000
H	0.000000000	4.520491000	0.055545000
H	0.000000000	4.549811000	2.683724000
H	0.000000000	4.586289000	-2.569032000
H	0.000000000	-2.641390000	4.574323000
H	0.000000000	-2.701141000	-4.513365000
H	0.000000000	-4.520491000	0.055545000
H	0.000000000	-4.549811000	2.683724000
H	0.000000000	-4.586289000	-2.569032000
H	0.865113000	0.000000000	-4.230053000
H	2.441676000	0.000000000	0.968718000
H	2.448348000	0.825111000	-0.460342000
H	2.448348000	-0.825111000	-0.460342000
H	-0.865113000	0.000000000	-4.230053000
H	-2.441676000	0.000000000	0.968718000
H	-2.448348000	0.825111000	-0.460342000
H	-2.448348000	-0.825111000	-0.460342000
N	0.000000000	1.447133000	1.485032000
N	0.000000000	1.461991000	-1.463788000
N	0.000000000	-1.447133000	1.485032000
N	0.000000000	-1.461991000	-1.463788000
N	2.091963000	0.000000000	0.014565000
N	-2.091963000	0.000000000	0.014565000

7. Ir[Phl](NH_3)$_2$ (anion), C_s; all-electron occupation (irrep α|β): a′ 77|77, a″ 53|52

Ir	0.011761000	-0.072251000	0.000000000
C	1.207741000	0.400059000	2.803124000
C	1.207741000	0.400059000	-2.803124000
C	2.474490000	0.538347000	3.459774000
C	2.474490000	0.538347000	-3.459774000
C	2.815724000	0.211594000	1.247633000
C	2.815724000	0.211594000	-1.247633000
C	3.442891000	0.162410000	0.000000000
C	3.455440000	0.402062000	2.511228000
C	3.455440000	0.402062000	-2.511228000
C	-0.043516000	0.442037000	3.399149000
C	-0.043516000	0.442037000	-3.399149000
C	-1.290752000	0.130009000	2.793750000
C	-1.290752000	0.130009000	-2.793750000
C	-2.555395000	0.093261000	3.405240000
C	-2.555395000	0.093261000	-3.405240000
C	-2.749984000	-0.590686000	1.271666000
C	-2.749984000	-0.590686000	-1.271666000
C	-3.224663000	-1.219882000	0.000000000

C	-3.469020000	-0.364106000	2.439246000
C	-3.469020000	-0.364106000	-2.439246000
H	0.058294000	-2.554452000	0.828901000
H	0.058294000	-2.554452000	-0.828901000
H	0.471877000	2.499378000	0.000000000
H	1.456066000	-2.235454000	0.000000000
H	2.597369000	0.707629000	4.518846000
H	2.597369000	0.707629000	-4.518846000
H	4.524192000	0.460249000	2.651839000
H	4.524192000	0.460249000	-2.651839000
H	4.524811000	0.198325000	0.000000000
H	-0.069286000	0.665254000	4.458220000
H	-0.069286000	0.665254000	-4.458220000
H	-0.932323000	2.208287000	0.829763000
H	-0.932323000	2.208287000	-0.829763000
H	-2.754842000	0.346940000	4.435059000
H	-2.754842000	0.346940000	-4.435059000
H	-2.916205000	-2.281180000	0.000000000
H	-4.315511000	-1.231475000	0.000000000
H	-4.526976000	-0.538822000	2.564799000
H	-4.526976000	-0.538822000	-2.564799000
N	0.446639000	-2.114734000	0.000000000
N	1.450174000	0.194655000	1.456228000
N	1.450174000	0.194655000	-1.456228000
N	-0.394585000	1.970785000	0.000000000
N	-1.432458000	-0.285036000	1.475482000
N	-1.432458000	-0.285036000	-1.475482000

8. Ir[Phl](NH$_3$)$_2$ (cation), C_s; all-electron occupation (irrep $\alpha|\beta$): a′ 77|76, a″ 52|52

Ir	0.009342000	-0.009137000	0.000000000
C	0.001385000	0.179464000	3.427074000
C	0.001385000	0.179464000	-3.427074000
C	1.237921000	0.285953000	2.808504000
C	1.237921000	0.285953000	-2.808504000
C	2.494825000	0.467857000	3.472573000
C	2.494825000	0.467857000	-3.472573000
C	2.799408000	0.383941000	1.243222000
C	2.799408000	0.383941000	-1.243222000
C	3.424489000	0.434328000	0.000000000
C	3.455808000	0.520534000	2.513348000
C	3.455808000	0.520534000	-2.513348000
C	-1.228602000	-0.046188000	2.829822000
C	-1.228602000	-0.046188000	-2.829822000
C	-2.476215000	-0.185266000	3.496328000
C	-2.476215000	-0.185266000	-3.496328000
C	-2.748258000	-0.476978000	1.290372000
C	-2.748258000	-0.476978000	-1.290372000

C	-3.403273000	-0.826753000	0.000000000
C	-3.419494000	-0.454342000	2.537074000
C	-3.419494000	-0.454342000	-2.537074000
H	0.543034000	2.577133000	0.000000000
H	1.360392000	-2.275787000	0.000000000
H	2.618383000	0.543431000	4.540388000
H	2.618383000	0.543431000	-4.540388000
H	4.498072000	0.558693000	0.000000000
H	4.517600000	0.649743000	2.642729000
H	4.517600000	0.649743000	-2.642729000
H	-0.002230000	0.266854000	4.505432000
H	-0.002230000	0.266854000	-4.505432000
H	-0.040309000	-2.521783000	0.822642000
H	-0.040309000	-2.521783000	-0.822642000
H	-0.861592000	2.346316000	0.822043000
H	-0.861592000	2.346316000	-0.822043000
H	-2.619808000	-0.097075000	4.560596000
H	-2.619808000	-0.097075000	-4.560596000
H	-3.571512000	-1.914612000	0.000000000
H	-4.410646000	-0.404291000	0.000000000
H	-4.474122000	-0.624510000	2.677164000
H	-4.474122000	-0.624510000	-2.677164000
N	0.362218000	-2.079314000	0.000000000
N	1.453043000	0.232857000	1.447751000
N	1.453043000	0.232857000	-1.447751000
N	-0.333864000	2.061960000	0.000000000
N	-1.436457000	-0.226681000	1.459199000
N	-1.436457000	-0.226681000	-1.459199000

9. Ir[Phl]$(NH_3)_2$ (triplet), C_s; all-electron occupation (irrep α|β): a′ 77|76, a″ 53|52

Ir	0.009507000	-0.020085000	0.000000000
C	1.236326000	0.348353000	2.802790000
C	1.236326000	0.348353000	-2.802790000
C	2.495427000	0.496309000	3.465779000
C	2.495427000	0.496309000	-3.465779000
C	2.824251000	0.306381000	1.246254000
C	2.824251000	0.306381000	-1.246254000
C	3.438520000	0.313408000	0.000000000
C	3.470613000	0.456388000	2.506067000
C	3.470613000	0.456388000	-2.506067000
C	-0.020950000	0.307895000	3.423568000
C	-0.020950000	0.307895000	-3.423568000
C	-1.250683000	0.030080000	2.820241000
C	-1.250683000	0.030080000	-2.820241000
C	-2.504792000	-0.079560000	3.471176000
C	-2.504792000	-0.079560000	-3.471176000

C	-2.755508000	-0.522232000	1.286349000
C	-2.755508000	-0.522232000	-1.286349000
C	-3.340561000	-0.994547000	0.000000000
C	-3.438181000	-0.430421000	2.506678000
C	-3.438181000	-0.430421000	-2.506678000
H	0.487638000	2.567135000	0.000000000
H	1.387885000	-2.254874000	0.000000000
H	2.617455000	0.608063000	4.530798000
H	2.617455000	0.608063000	-4.530798000
H	4.518703000	0.380448000	0.000000000
H	4.536826000	0.541891000	2.640904000
H	4.536826000	0.541891000	-2.640904000
H	-0.016063000	-2.513306000	0.826046000
H	-0.016063000	-2.513306000	-0.826046000
H	-0.033815000	0.461719000	4.493690000
H	-0.033815000	0.461719000	-4.493690000
H	-0.913841000	2.287681000	0.825632000
H	-0.913841000	2.287681000	-0.825632000
H	-2.669125000	0.079236000	4.524437000
H	-2.669125000	0.079236000	-4.524437000
H	-3.292154000	-2.095982000	0.000000000
H	-4.406810000	-0.764438000	0.000000000
H	-4.492150000	-0.610303000	2.644574000
H	-4.492150000	-0.610303000	-2.644574000
N	0.386527000	-2.078737000	0.000000000
N	1.453820000	0.234873000	1.457596000
N	1.453820000	0.234873000	-1.457596000
N	-0.376990000	2.033825000	0.000000000
N	-1.435330000	-0.240882000	1.468450000
N	-1.435330000	-0.240882000	-1.468450000

10. Au[Cor], C_{2v}; all-electron occupation (irrep α|β): a_1 51|51, a_2 10|10, b_1 39|39, b_2 16|16

Au	0.000000000	0.000000000	0.012268000
C	0.000000000	0.000000000	3.323129000
C	0.714396000	0.000000000	-2.741324000
C	1.254304000	0.000000000	2.700361000
C	1.826511000	0.000000000	-3.624198000
C	2.563850000	0.000000000	3.291367000
C	2.609205000	0.000000000	-1.474923000
C	2.791167000	0.000000000	1.024526000
C	2.981971000	0.000000000	-2.856193000
C	3.330966000	0.000000000	-0.267103000
C	3.486161000	0.000000000	2.282333000
C	-0.714396000	0.000000000	-2.741324000
C	-1.254304000	0.000000000	2.700361000
C	-1.826511000	0.000000000	-3.624198000
C	-2.563850000	0.000000000	3.291367000

C	-2.609205000	0.000000000	-1.474923000
C	-2.791167000	0.000000000	1.024526000
C	-2.981971000	0.000000000	-2.856193000
C	-3.330966000	0.000000000	-0.267103000
C	-3.486161000	0.000000000	2.282333000
H	0.000000000	0.000000000	4.403754000
H	1.775519000	0.000000000	-4.699987000
H	2.757368000	0.000000000	4.351318000
H	3.994007000	0.000000000	-3.225737000
H	4.409602000	0.000000000	-0.334356000
H	4.559206000	0.000000000	2.383591000
H	-1.775519000	0.000000000	-4.699987000
H	-2.757368000	0.000000000	4.351318000
H	-3.994007000	0.000000000	-3.225737000
H	-4.409602000	0.000000000	-0.334356000
H	-4.559206000	0.000000000	2.383591000
N	1.250004000	0.000000000	-1.480386000
N	1.447303000	0.000000000	1.345420000
N	-1.250004000	0.000000000	-1.480386000
N	-1.447303000	0.000000000	1.345420000

11. Au[Cor] anion, C_{2v}; all-electron occupation (irrep α|β): a_1 51|51, a_2 11|10, b_1 39|39, b_2 16|16

Au	0.000000000	0.000000000	0.002640000
C	0.000000000	0.000000000	3.331353000
C	0.724587000	0.000000000	-2.762679000
C	1.256180000	0.000000000	2.712930000
C	1.817787000	0.000000000	-3.637025000
C	2.556716000	0.000000000	3.300920000
C	2.621343000	0.000000000	-1.489869000
C	2.799086000	0.000000000	1.027484000
C	2.995174000	0.000000000	-2.854688000
C	3.348380000	0.000000000	-0.261322000
C	3.488872000	0.000000000	2.288974000
C	-0.724587000	0.000000000	-2.762679000
C	-1.256180000	0.000000000	2.712930000
C	-1.817787000	0.000000000	-3.637025000
C	-2.556716000	0.000000000	3.300920000
C	-2.621343000	0.000000000	-1.489869000
C	-2.799086000	0.000000000	1.027484000
C	-2.995174000	0.000000000	-2.854688000
C	-3.348380000	0.000000000	-0.261322000
C	-3.488872000	0.000000000	2.288974000
H	0.000000000	0.000000000	4.413699000
H	1.766813000	0.000000000	-4.713888000
H	2.748429000	0.000000000	4.362753000
H	4.008337000	0.000000000	-3.224339000
H	4.427766000	0.000000000	-0.324309000
H	4.562633000	0.000000000	2.393663000

H	-1.766813000	0.000000000	-4.713888000
H	-2.748429000	0.000000000	4.362753000
H	-4.008337000	0.000000000	-3.224339000
H	-4.427766000	0.000000000	-0.324309000
H	-4.562633000	0.000000000	2.393663000
N	1.259912000	0.000000000	-1.493340000
N	1.454181000	0.000000000	1.345174000
N	-1.259912000	0.000000000	-1.493340000
N	-1.454181000	0.000000000	1.345174000

12. Au[Cor] cation, C_{2v}; all-electron occupation (irrep α|β): a_1 51|51, a_2 10|10, b_1 39|39, b_2 16|15

Au	0.000000000	0.000000000	0.014000000
C	0.000000000	0.000000000	3.322798000
C	0.726827000	0.000000000	-2.730849000
C	1.262743000	0.000000000	2.704743000
C	1.833187000	0.000000000	-3.626271000
C	2.563911000	0.000000000	3.297246000
C	2.613694000	0.000000000	-1.475427000
C	2.782278000	0.000000000	1.037390000
C	2.986950000	0.000000000	-2.863337000
C	3.325629000	0.000000000	-0.277882000
C	3.487687000	0.000000000	2.281062000
C	-0.726827000	0.000000000	-2.730849000
C	-1.262743000	0.000000000	2.704743000
C	-1.833187000	0.000000000	-3.626271000
C	-2.563911000	0.000000000	3.297246000
C	-2.613694000	0.000000000	-1.475427000
C	-2.782278000	0.000000000	1.037390000
C	-2.986950000	0.000000000	-2.863337000
C	-3.325629000	0.000000000	-0.277882000
C	-3.487687000	0.000000000	2.281062000
H	0.000000000	0.000000000	4.404427000
H	1.776199000	0.000000000	-4.701400000
H	2.761540000	0.000000000	4.356244000
H	3.998436000	0.000000000	-3.234354000
H	4.405212000	0.000000000	-0.340525000
H	4.560614000	0.000000000	2.379579000
H	-1.776199000	0.000000000	-4.701400000
H	-2.761540000	0.000000000	4.356244000
H	-3.998436000	0.000000000	-3.234354000
H	-4.405212000	0.000000000	-0.340525000
H	-4.560614000	0.000000000	2.379579000
N	1.247963000	0.000000000	-1.480506000
N	1.449059000	0.000000000	1.347958000
N	-1.247963000	0.000000000	-1.480506000
N	-1.449059000	0.000000000	1.347958000

13. AuCor (triplet), C_{2v}; all-electron occupation (irrep α|β): a_1 51|51, a_2 11|10, b_1 39|39, b_2 16|15

Au	0.000000000	0.000000000	-0.000656000
C	0.000000000	0.000000000	3.321053000
C	0.735822000	0.000000000	-2.756481000
C	1.261093000	0.000000000	2.712586000
C	1.822225000	0.000000000	-3.635730000
C	2.553767000	0.000000000	3.304810000
C	2.620616000	0.000000000	-1.487296000
C	2.786543000	0.000000000	1.041945000
C	2.996174000	0.000000000	-2.855901000
C	3.342122000	0.000000000	-0.272538000
C	3.485534000	0.000000000	2.290518000
C	-0.735822000	0.000000000	-2.756481000
C	-1.261093000	0.000000000	2.712586000
C	-1.822225000	0.000000000	-3.635730000
C	-2.553767000	0.000000000	3.304810000
C	-2.620616000	0.000000000	-1.487296000
C	-2.786543000	0.000000000	1.041945000
C	-2.996174000	0.000000000	-2.855901000
C	-3.342122000	0.000000000	-0.272538000
C	-3.485534000	0.000000000	2.290518000
H	0.000000000	0.000000000	4.403367000
H	1.769336000	0.000000000	-4.710949000
H	2.746286000	0.000000000	4.365248000
H	4.008451000	0.000000000	-3.224333000
H	4.420583000	0.000000000	-0.331622000
H	4.558099000	0.000000000	2.391670000
H	-1.769336000	0.000000000	-4.710949000
H	-2.746286000	0.000000000	4.365248000
H	-4.008451000	0.000000000	-3.224333000
H	-4.420583000	0.000000000	-0.331622000
H	-4.558099000	0.000000000	2.391670000
N	1.256623000	0.000000000	-1.492585000
N	1.456726000	0.000000000	1.343059000
N	-1.256623000	0.000000000	-1.492585000
N	-1.456726000	0.000000000	1.343059000

14. Pt[Por], D_{4h}; all-electron occupation (irrep α|β): a_{1g} 19|19, a_{2g} 6|6, b_{1g} 11|11, b_{2g} 11|11, e_g 12|12, a_{1u} 1|1, a_{2u} 3|3, b_{2u} 2|2, e_u 46|46

Pt	0.000000000	0.000000000	0.000000000
C	0.000000000	3.418980000	0.000000000
C	0.000000000	-3.418980000	0.000000000
C	1.233074000	2.792923000	0.000000000
C	1.233074000	-2.792923000	0.000000000
C	2.505854000	3.463578000	0.000000000

C	2.505854000	-3.463578000	0.000000000
C	2.792923000	1.233074000	0.000000000
C	2.792923000	-1.233074000	0.000000000
C	3.418980000	0.000000000	0.000000000
C	3.463578000	2.505854000	0.000000000
C	3.463578000	-2.505854000	0.000000000
C	-1.233074000	2.792923000	0.000000000
C	-1.233074000	-2.792923000	0.000000000
C	-2.505854000	3.463578000	0.000000000
C	-2.505854000	-3.463578000	0.000000000
C	-2.792923000	1.233074000	0.000000000
C	-2.792923000	-1.233074000	0.000000000
C	-3.418980000	0.000000000	0.000000000
C	-3.463578000	2.505854000	0.000000000
C	-3.463578000	-2.505854000	0.000000000
H	0.000000000	4.500158000	0.000000000
H	0.000000000	-4.500158000	0.000000000
H	2.631440000	4.533874000	0.000000000
H	2.631440000	-4.533874000	0.000000000
H	4.500158000	0.000000000	0.000000000
H	4.533874000	2.631440000	0.000000000
H	4.533874000	-2.631440000	0.000000000
H	-2.631440000	4.533874000	0.000000000
H	-2.631440000	-4.533874000	0.000000000
H	-4.500158000	0.000000000	0.000000000
H	-4.533874000	2.631440000	0.000000000
H	-4.533874000	-2.631440000	0.000000000
N	1.436174000	1.436174000	0.000000000
N	1.436174000	-1.436174000	0.000000000
N	-1.436174000	1.436174000	0.000000000
N	-1.436174000	-1.436174000	0.000000000

15. Pt[Por] anion, C_s; all-electron occupation (irrep α|β): a′ 70|70, a″ 50|49

Pt	-0.000047000	-0.000483000	0.000000000
C	0.673026000	-0.067520000	4.234866000
C	0.673026000	-0.067520000	-4.234866000
C	1.106054000	-0.110901000	2.856853000
C	1.106054000	-0.110901000	-2.856853000
C	2.406561000	-0.240322000	2.438516000
C	2.406561000	-0.240322000	-2.438516000
C	2.851808000	-0.284399000	1.107283000
C	2.851808000	-0.284399000	-1.107283000
C	4.200543000	-0.417479000	0.689400000
C	4.200543000	-0.417479000	-0.689400000
C	-0.673129000	0.066533000	4.234864000
C	-0.673129000	0.066533000	-4.234864000
C	-1.106123000	0.110206000	2.856851000

C	-1.106123000	0.110206000	-2.856851000
C	-2.406578000	0.240143000	2.438515000
C	-2.406578000	0.240143000	-2.438515000
C	-2.851781000	0.284653000	1.107283000
C	-2.851781000	0.284653000	-1.107283000
C	-4.200409000	0.418802000	0.689401000
C	-4.200409000	0.418802000	-0.689401000
H	1.337806000	-0.133618000	5.082051000
H	1.337806000	-0.133618000	-5.082051000
H	3.165383000	-0.315752000	3.206180000
H	3.165383000	-0.315752000	-3.206180000
H	5.044034000	-0.500270000	1.356387000
H	5.044034000	-0.500270000	-1.356387000
H	-1.337912000	0.132616000	5.082047000
H	-1.337912000	0.132616000	-5.082047000
H	-3.165371000	0.315854000	3.206180000
H	-3.165371000	0.315854000	-3.206180000
H	-5.043841000	0.502189000	1.356390000
H	-5.043841000	0.502189000	-1.356390000
N	2.036311000	-0.203749000	0.000000000
N	-0.000041000	-0.000417000	2.043633000
N	-0.000041000	-0.000417000	-2.043633000
N	-2.036325000	0.203593000	0.000000000

16. Pt[Pot] cation, C_s; all-electron occupation (irrep α|β): a′ 93|93, a″ 26|25

Pt	-0.000009000	0.000038000	0.000000000
C	0.007160000	3.418105000	0.000000000
C	1.227707000	2.795148000	0.000000000
C	1.234681000	-2.777900000	0.000000000
C	2.515114000	-3.462639000	0.000000000
C	2.515662000	3.469455000	0.000000000
C	2.777828000	1.234601000	0.000000000
C	2.795215000	-1.227804000	0.000000000
C	3.418075000	-0.007209000	0.000000000
C	3.462583000	2.515132000	0.000000000
C	3.469446000	-2.515756000	0.000000000
C	-0.007138000	-3.418039000	0.000000000
C	-1.227685000	-2.795107000	0.000000000
C	-1.234703000	2.777959000	0.000000000
C	-2.515220000	3.462651000	0.000000000
C	-2.515599000	-3.469457000	0.000000000
C	-2.777837000	-1.234637000	0.000000000
C	-2.795238000	1.227788000	0.000000000
C	-3.418092000	0.007173000	0.000000000
C	-3.462541000	-2.515131000	0.000000000
C	-3.469522000	2.515702000	0.000000000
H	0.000616000	4.498564000	0.000000000
H	2.632543000	-4.533398000	0.000000000

H	2.641717000	4.539308000	0.000000000
H	4.498531000	-0.000632000	0.000000000
H	4.533308000	2.632938000	0.000000000
H	4.539338000	-2.641462000	0.000000000
H	-0.000535000	-4.498493000	0.000000000
H	-2.632628000	4.533422000	0.000000000
H	-2.641566000	-4.539312000	0.000000000
H	-4.498553000	0.000590000	0.000000000
H	-4.533250000	-2.632981000	0.000000000
H	-4.539414000	2.641315000	0.000000000
N	1.429980000	-1.434012000	0.000000000
N	1.434014000	1.429927000	0.000000000
N	-1.430012000	1.434074000	0.000000000
N	-1.433983000	-1.429919000	0.000000000

17. Pt[Por] (triplet), C_s; all-electron occupation (irrep α|β): a′ 93|93, a″ 27|25

Pt	-0.000058000	-0.000079000	0.000000000
C	0.026200000	3.431535000	0.000000000
C	1.240000000	2.816647000	0.000000000
C	1.254348000	-2.793673000	0.000000000
C	2.499098000	-3.466589000	0.000000000
C	2.524661000	3.481653000	0.000000000
C	2.793612000	1.245435000	0.000000000
C	2.812805000	-1.241236000	0.000000000
C	3.427253000	0.006991000	0.000000000
C	3.473507000	2.521801000	0.000000000
C	3.475517000	-2.496578000	0.000000000
C	-0.026193000	-3.431632000	0.000000000
C	-1.240030000	-2.816778000	0.000000000
C	-1.254306000	2.793600000	0.000000000
C	-2.499028000	3.466586000	0.000000000
C	-2.524669000	-3.481635000	0.000000000
C	-2.793668000	-1.245416000	0.000000000
C	-2.812847000	1.241246000	0.000000000
C	-3.427287000	-0.006966000	0.000000000
C	-3.473524000	-2.521694000	0.000000000
C	-3.475479000	2.496633000	0.000000000
H	0.019738000	4.512774000	0.000000000
H	2.623676000	-4.536498000	0.000000000
H	2.656095000	4.551214000	0.000000000
H	4.508458000	0.015876000	0.000000000
H	4.544531000	2.641247000	0.000000000
H	4.544751000	-2.628267000	0.000000000
H	-0.019687000	-4.512865000	0.000000000
H	-2.623527000	4.536509000	0.000000000
H	-2.656191000	-4.551181000	0.000000000
H	-4.508495000	-0.015858000	0.000000000
H	-4.544558000	-2.641048000	0.000000000

H	-4.544701000	2.628404000	0.000000000
N	1.442919000	-1.447203000	0.000000000
N	1.449635000	1.444826000	0.000000000
N	-1.442902000	1.447122000	0.000000000
N	-1.449660000	-1.444902000	0.000000000
